# Expanding repertoire of SARS-CoV-2 deletion mutations contributes to evolution of highly transmissible variants

**DOI:** 10.1038/s41598-022-26646-5

**Published:** 2023-01-05

**Authors:** A. J. Venkatakrishnan, Praveen Anand, Patrick J. Lenehan, Pritha Ghosh, Rohit Suratekar, Eli Silvert, Colin Pawlowski, Abhishek Siroha, Dibyendu Roy Chowdhury, John C. O’Horo, Joseph D. Yao, Bobbi S. Pritt, Andrew P. Norgan, Ryan T. Hurt, Andrew D. Badley, John Halamka, Venky Soundararajan

**Affiliations:** 1 nference, Cambridge, MA 02139 USA; 2 nference Labs, Bengaluru, Karnataka India; 3 Mayo Clinic, Rochester, MN 55902 USA; 4 Anumana, Cambridge, MA 02139 USA

**Keywords:** Viral infection, Molecular evolution

## Abstract

The emergence of highly transmissible SARS-CoV-2 variants and vaccine breakthrough infections globally mandated the characterization of the immuno-evasive features of SARS-CoV-2. Here, we systematically analyzed 2.13 million SARS-CoV-2 genomes from 188 countries/territories (up to June 2021) and performed whole-genome viral sequencing from 102 COVID-19 patients, including 43 vaccine breakthrough infections. We identified 92 Spike protein mutations that increased in prevalence during at least one surge in SARS-CoV-2 test positivity in any country over a 3-month window. Deletions in the Spike protein N-terminal domain were highly enriched for these ‘*surge-associated mutations*’ (Odds Ratio = 14.19, 95% CI 6.15–32.75, *p* value = 3.41 × 10^–10^). Based on a longitudinal analysis of mutational prevalence globally, we found an expanding repertoire of Spike protein deletions proximal to an antigenic supersite in the N-terminal domain that may be one of the key contributors to the evolution of highly transmissible variants. Finally, we generated clinically annotated SARS-CoV-2 whole genome sequences from 102 patients and identified 107 unique mutations, including 78 substitutions and 29 deletions. In five patients, we identified distinct deletions between residues 85–90, which reside within a linear B cell epitope. Deletions in this region arose contemporaneously on a diverse background of variants across the globe since December 2020. Overall, our findings based on genomic-epidemiology and clinical surveillance suggest that the genomic deletion of dispensable antigenic regions in SARS-CoV-2 may contribute to the evasion of immune responses and the evolution of highly transmissible variants.

## Introduction

The COVID-19 pandemic killed millions of individuals worldwide^1^. The continual emergence of SARS-CoV-2 variants with increased transmissibility and capacity for immune escape (e.g., Delta, Omicron) threatens to prolong the pandemic and drive devastating outbreaks^2^. While multiple vaccines have demonstrated high effectiveness in clinical trials and real world studies^3–5^, it is known that vaccine breakthrough infections can occur, even in individuals with robust neutralizing antibody responses^6,7^. Variant classification schemes were developed by the U.S. Centers for Disease Control and Prevention (CDC)^8^ and the World Health Organisation (WHO)^9^ based on factors such as prevalence, evidence of transmissibility and disease severity, and ability to be neutralized by existing therapeutics or sera from vaccinated patients. Early and rapid detection of emerging Variants of Concern/Interest is imperative to combat and contain future outbreaks.

Mapping the mutational landscape of SARS-CoV-2 in the context of natural and vaccine-induced immune responses is critical to understand the virus’s molecular strategies for immune evasion. To this end, neutralizing antibodies which target the receptor-binding domain (RBD) or the N-terminal domain (NTD) of the Spike protein have been isolated from the sera of COVID-19 patients^10–12^. Recent studies also found that several neutralizing antibodies target a single antigenic supersite in the NTD of the Spike protein^13,14^. The NTD is also a hotspot for in-frame deletions in the SARS-CoV-2 genome, with four recurrent deletion regions (RDRs) identified^15^. Several such deletions have been experimentally demonstrated to reduce neutralization by NTD-targeting neutralizing antibodies^13,15^. Whether additional deletions are emerging in SARS-CoV-2 variants that drive case surges or vaccine breakthrough infections needs to be determined.

**Figure 1:**
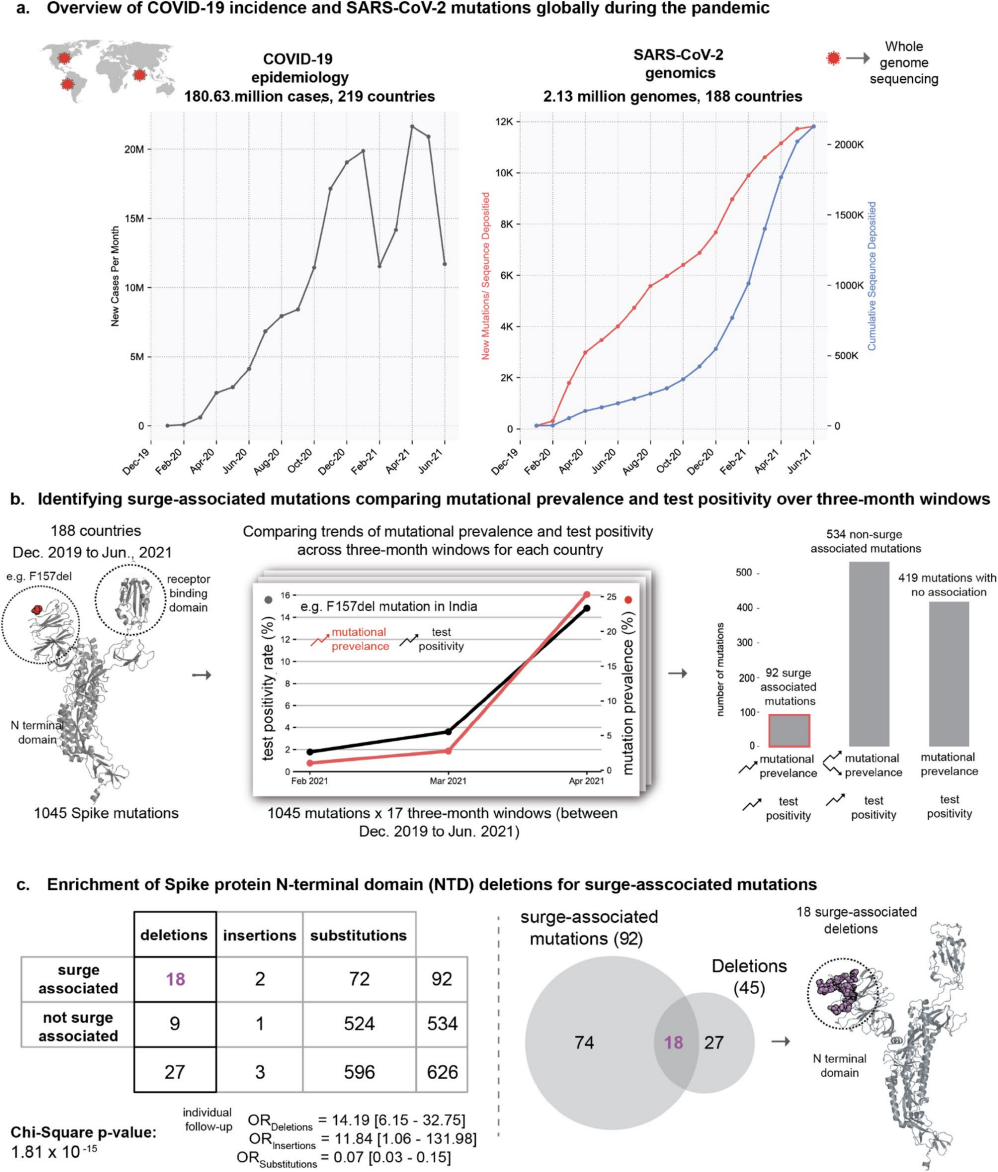
Identifying potential SARS-CoV-2 variants contributing to COVID-19 surges. (**a**) Overview of COVID-19 incidence and accumulation of SARS-CoV-2 mutations globally during the pandemic. (**b**) Identification of surge-associated mutations by comparing mutational prevalence and test positivity over 3-month windows. Structure shows the sites of all 1045 mutations (as red spheres) on a single subunit of the homo-trimeric Spike protein that are present in at least 100 GISAID entries each site can harbor multiple mutations. Line graph shows correlation between trends of mutational prevalence (e.g. ΔF157) and test positivity during a 3-month window (e.g. February 2021 to April 2021 in India). These correlations are computed for all 3-month windows across all the countries being studied. Bar plot shows the frequency of mutations that fall in each combination of trends: (1) 92 surge-associated mutations: mutational prevalence and test positivity are increasing monotonically, (2) 534 non-surge associated mutations: detected during test positivity surges but without a monotonic increase in prevalence, and (3) 419 mutations for which no associations could be made between mutational prevalence and  the test positivity trend. (**c**) Mutations detected in the time periods associated with surge in PCR positivity. In total, 626 mutations (92 + 534) are associated with an increasing positivity trend, and the enrichments of different types of mutations (deletions, insertions and substitutions) are shown. The 2 × 3 contingency table shows the distribution of mutations based on surge-association and mutation category (deletion, insertion, substitution). Chi-square test results are also shown. Venn diagram shows the overlap of surge-associated mutations and all the deletion mutations. Structure shows the sites of all 18 surge-associated deletion mutations (as magenta spheres) on a single subunit of the Spike protein localized within the N-terminal domain. The structural rendering was made using a three-dimensional structure obtained from the Protein Data Bank (PDB identifier: 6VSB).

Since the beginning of the pandemic, concerted global data-sharing efforts led to the rapid development of large-scale genomic and epidemiological COVID-19 resources. Around 2.13 million SARS-CoV-2 genomes from 188 countries/territories were deposited in the GISAID database (as of 30 June 2021)^16^ (Fig. 1). In addition, we performed whole-genome sequencing of SARS-CoV-2 from 102 COVID-19 patients at the Mayo Clinic who required hospitalization or were categorized as vaccine breakthrough infections. On the epidemiology front, population-level metrics including SARS-CoV-2 test positivity rates are being collected in databases such as Our World in Data (OWID)^17^. Such unprecedented availability of genomic-epidemiological data combined with patient-level clinical genomic data provides a timely opportunity to systematically characterize the immuno-evasive features of SARS-CoV-2.

In this study, we uncovered that deletion mutations in the Spike protein have a high likelihood of being associated with surges in community transmission. Further, based on a global longitudinal analysis of deletions, we also highlight that the repertoire of deletion-prone regions of the Spike protein expanded during the pandemic, pointing to an evolutionary strategy of “antigenic minimalism” to evade immune responses. Finally, using whole genome sequencing linked to clinical annotations derived from electronic health records, we also identified an emerging hotspot of deletion mutations in SARS-CoV-2 isolated from hospitalized COVID-19 patients or vaccine-breakthrough infections. These deletions were distinct from the background set of mutations observed in the geographical region and mapped to a previously characterized linear B-cell epitope, thus representing candidates to be monitored for escape mutations.

## Results

### Deletions were enriched for association with surges in community transmission of SARS-CoV-2

Analysis of 2,128,574 SARS-CoV-2 genome sequences obtained from the GISAID database^16^ (Fig. 1a) revealed 1045 amino acid mutations (missense, insertions and deletions) in the Spike protein which were present in at least 100 deposited sequences (Figure S1). This included 995 substitutions (95.21%), 45 deletions (4.3%), and 5 insertions (0.48%). To identify the mutations associated with surges in the community transmission of SARS-CoV-2 (“surge-associated mutations''), we shortlisted mutations that increased in prevalence monotonically during 3-month periods of monotonically increasing test positivity (Fig. 1b). We identified 92 such surge-associated mutations and found that this approach recapitulated 42 out of 59 (71%) mutations known to be present in the CDC variants of interest or concern, including D614G, E484K, N501Y, P681H, P681R, ΔH69/V70, and ΔY144.

Further, we investigated whether a class of mutations (missense, insertions, and/or deletions) is enriched for association with surges. Interestingly, we found that deletions were associated with surges more frequently than expected by chance (Table S1). Specifically, 18 of 45 (40%) deletions were associated with one or more surges, compared to only 74 of 599 non-deletion mutations (12%) (Fig. 1c; Odds Ratio = 14.19, 95% CI 6.15–32.75, *p* value = 3.41 × 10^–10^). These surge-associated deletions in the Spike protein occur exclusively in the N-terminal domain (NTD), which is interesting considering the recurrent deletion regions (RDRs) in the N-terminal domain^15^. This raises the possibility that the acquisition of deletion mutations in the NTD and substitutions in functionally important regions (e.g., L452R in the receptor binding domain) may contribute to the evolution of highly transmissible variants.

### Expansion of deletable regions in a Spike protein antigenic supersite coincided with mass vaccination

**Figure 2:**
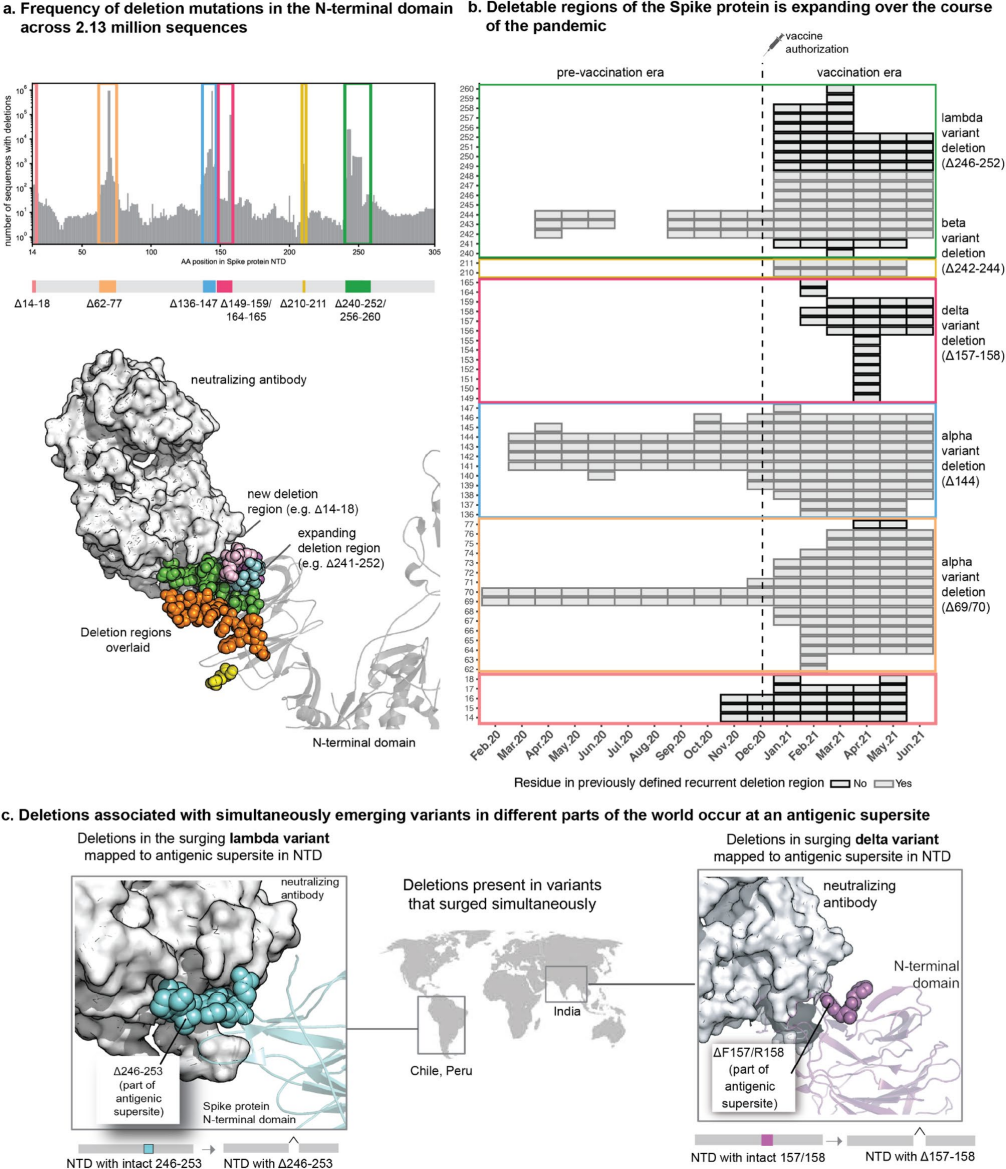
The repertoire of deletions in the Spike protein N-terminal domain is expanding over the course of the pandemic. (**a**) Frequency of occurrences of deletion mutations in the N-terminal domain across 2.13 million Spike protein sequences (as of 30 June 2021). The recurrent deletion regions, both known and new, are illustrated schematically and mapped on the structure of the Spike protein. Positions corresponding to the deletion mutations in the Spike protein are shown as colored spheres, and the neutralizing antibody is shown using a surface representation in grey. (**b**) Heatmap showing the expansion of “deletable” regions in the course of the pandemic, where the rows denote residue positions in the Spike protein and columns denote the time course of the pandemic (in months). Each box denotes the frequency of a given deletion mutation across the world in that month. The color of the boxes corresponds to a frequency of 1 to 100,000 sequences shown on a log10 scale. (**c**) Sites of deletion mutations associated with surges in different parts of the world are shown as spheres on the 3D structure of the Spike protein complexed with neutralizing antibody 4A8 (PDB identifier: 7C2L described by Chi et al.^18^, retrieved from the PDB).

To understand how the landscape of Spike protein deletions evolved during the COVID-19 pandemic, we assessed the prevalence of deletions at each residue among deposited sequences monthly (see Methods, Table S2). We identified seven regions within the NTD in which deletions are observed at rates significantly higher than the background frequency (Fig. 2a). One of these regions (residues 240–252) appeared to have expanded since the previous study defining Spike protein recurrent deletion regions^15^, and three of them (residues 14–18, 149–159 and 256–260) were not present at the time of this prior analysis. The remaining three (residues 62–77, 136–147, and 210–211) were consistent with previously identified recurrent deletion regions. The regions which were frequently deleted during the early months (residues 69–70, 141–144, and 242–244) remained prevalent subsequently, with Δ69/70 and Δ144 present in the Alpha variant and Δ242–244 present in the Beta variant (Fig. 2b, Figure S2).

Importantly, most of these recurrent deletion regions mapped to an antigenic supersite which is bound by previously isolated NTD-targeting neutralizing antibodies (Fig. 2a,c)^13–15^, suggesting a common evolutionary trend of antigenic minimalism by which SARS-CoV-2 evolves to discard residues which are likely to be targeted by host immune responses. Thus, the expansion and emergence of new deletable regions during the months in which mass vaccination campaigns were implemented across the globe is intriguing from an immunological perspective. The Δ246–252 deletion, which can be seen as an expansion of the previously observed Δ242–244, was uniquely present in the Lambda variant that surged in Peru and Chile in early 2021 (Fig. 2c). Experimental evidence from Chile suggested that the Lambda variant was less susceptible to neutralization by sera from individuals who received the CoronaVac vaccine^19^. Along with several other substitution mutations, the Δ156–158 deletion was a defining mutation of the Delta variant which drove substantial case surges elsewhere. Experimental evidence also showed reduced neutralization of this variant by sera from convalescent COVID-19 patients and vaccinated individuals^20–23^.

No strains carrying deletions in residues 14–18 were labeled as variants of interest or concern, consistent with their lack of association with PCR positivity surges during the study period. However, it is important to note that several months can pass between the emergence of an RDR and its association with case surges. Indeed, deletions at residues 141–144, 242–244, and 69–70 were not associated with surges until 5, 9, and 10 months after the first emergence of these RDRs, respectively (Figure S2). Thus, it is important to monitor circulating variants and identify new deletable regions as they emerge.

### Whole-genome sequencing of clinically annotated SARS-CoV-2 samples showed a novel emerging deletion region in a linear B cell epitope

**Figure 3:**
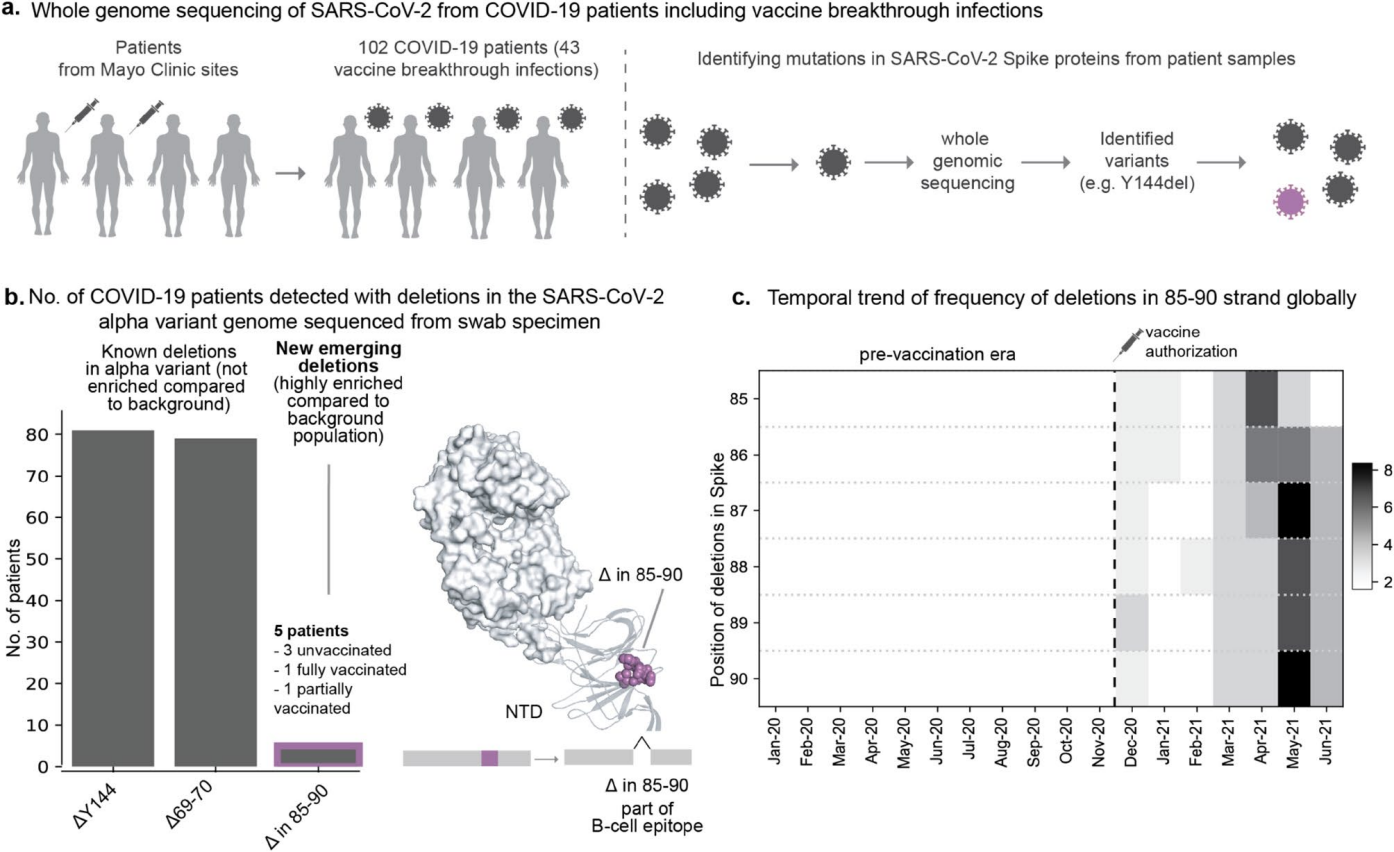
New deletion mutations present in the Spike proteins sequences derived from COVID-19 patients including vaccinated patients. (**a**) Whole genome sequencing of SARS-CoV-2 isolated from hospitalized COVID-19 patients or vaccination breakthroughs/reinfections from Mayo Clinic. (**b**) Barplot showing comparison of frequencies of deletions in patients infected with SARS-CoV-2 Alpha variant. New group of emerging deletions in the 85–90 region that is highly enriched compared to the background population is highlighted. The deletions in the other rarer lineages (e.g. Gamma variant) are not shown for clarity. The site of the emerging deletion mutations in patients is shown as spheres on the 3D structure of the Spike protein complexed with neutralizing antibody 4A8 (PDB identifier: 7C2L described by Chi et al.^18^, retrieved from the PDB). (**c**) Heatmap displaying temporal trend of increasing frequency of deletions corresponding to 85–90 region based on global SARS-CoV-2 genomic data from the GISAID database. The columns correspond to months and rows correspond to positions with deletions in the 85–90 region. The frequency of the mutations is mapped to a color scale of white to black.

The genomic-epidemiology analysis presented above based on publicly accessible data suggested that SARS-CoV-2 acquires deletion mutations to evade neutralizing antibodies and that the deletable regions are expanding. However, the genome sequences deposited in publicly accessible databases (e.g., GISAID) lacked clinical and phenotypic annotations such as vaccination status and disease severity of the corresponding COVID-19 patients. To address this, we performed whole genome viral sequencing from 102 COVID-19 patients at the Mayo Clinic health system, for whom we have complete longitudinal health records and vaccination history (Fig. 3a, Table S3). Of these, 41 cases were hospitalized for COVID-19, 43 cases were post-vaccine infections, and 2 cases were reinfections or persistent infections. In total, we identified 107 unique mutations, of which 29 are deletions. All observed Spike protein deletions in this cohort occurred in the NTD, with the Alpha variant (containing Δ144 and ΔH69/V70) showing a prevalence of nearly 80%.

Although diverse deletion profiles in SARS-CoV-2 genomes were observed in COVID-19 patients^24^, we identified a new deletion hotpot spanning residues 85–90 in five patients infected with the Alpha variant (Fig. 3b). Four of these patients were unvaccinated and hospitalized, while one was a vaccine breakthrough case. Clinical characterization based on EHR notes showed no distinct pre-existing conditions or complications enriched among these five patients (Table S4). It is noteworthy that no other genomes deposited in GISAID from Minnesota (n = 22,166) between March 1, 2021 and June 1, 2021 contained any deletions in this region (Odds ratio: 2501, 95% CI 137–45,539, *p* value < 0.0001). On the other hand, deletions in this region have emerged in the context of various lineages (e.g., Alpha and Gamma) across the world since December 2020 (Fig. 3c, Table S5). While these residues are not part of the antigenic supersite (Fig. 3b), a linear peptide containing these amino acids was previously identified as a linear B cell epitope bound by COVID-19 patient-derived antibodies^25^. The contemporaneous independent emergence of these deletions suggests an evolutionary pressure acting at this region of the Spike protein.

## Discussion

**Figure 4:**
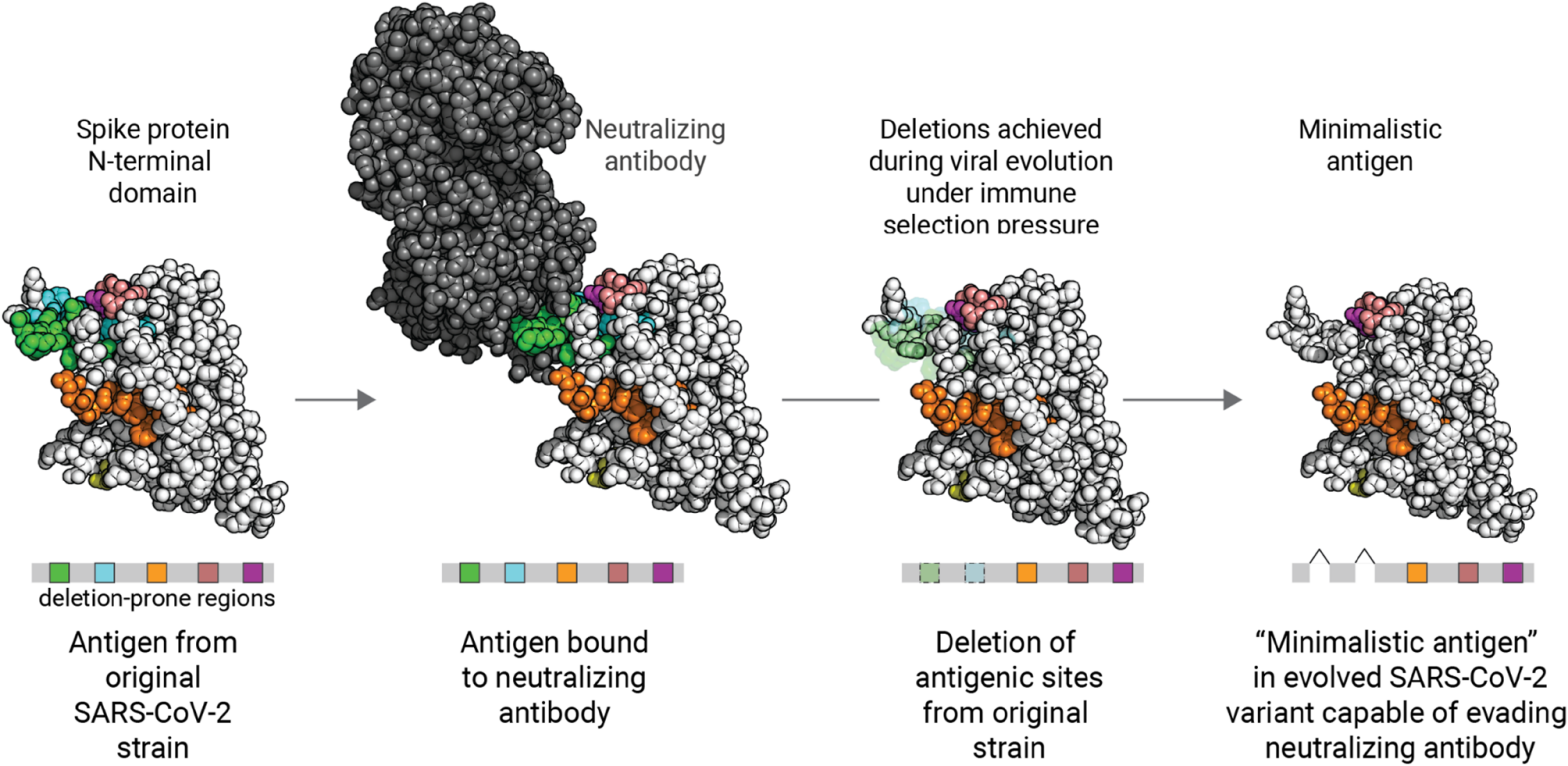
Schematic overview of the Spike protein N-terminal domain acquiring deletion mutations during evolution to evade immune responses. The deletion mutations occur concurrently with other substitution mutations (not highlighted) in the background. The schematic was made using a three-dimensional structure obtained from the Protein Data Bank (PDB identifier: 7C2L described by Chi et al.).^18^.

The worldwide mass vaccination campaign has had a profound impact on COVID-19 transmission. However, certain variants are less susceptible to neutralization by sera from vaccinated individuals and convalescent COVID-19 patients^26,27^. Such findings motivate the need to vigilantly track the emergence of new variants and to determine whether they are likely to cause surges or vaccine breakthrough infections. Here, through an integrated analysis of genomic, epidemiologic, and patient-level clinical data, we found that (1) deletions are strongly associated with surges in community transmission (Fig. 1), (2) the repertoire of deletions in the Spike protein expanded over the course of the pandemic coinciding with mass vaccination (Fig. 2), and (3) a novel deletion hotspot emerged in a B-cell epitope (Fig. 3). Importantly, deletion mutations do not operate independently of other mutation classes. In addition to deletion mutations, several substitution mutations are also associated with surges in cases (e.g., L452R and T478K in the receptor-binding domain). Substitutions have been more common compared to insertion/deletion events (indels), as noted in previous studies^28,29^ and confirmed by us here (Figure S4). Thus, a concerted evolution of strategically placed deletions and substitutions appears to confer SARS-CoV-2 with improved fitness to evade immunity and achieve efficient transmission between hosts (Fig. 4). Deletions could also conceivably alter fitness or persistence by influencing steps in the viral life cycle such as RNA packaging^24,30^.

Our finding that Spike protein NTD deletions were strongly enriched for association with test positivity surges is notable in the context of a previous report identifying the NTD as the most common site of deletions^15^. Specifically, this prior study highlighted four recurrent deletion regions in the NTD based on the GISAID data deposited as of October 2020 (146,795 total sequences). Several of these regions overlap with the residues of the recently identified NTD antigenic supersite, and deletions within them can abrogate binding to neutralizing antibodies^13–15^. Our findings build upon this prior work by examining the deletions which have arisen in the interim, during which over 1.5 million additional sequences have been deposited. In addition to validating the previously suggested definitions of recurrent deletion regions RDR1 (ΔH69/V70 and flanking deletions), RDR2 (ΔY144 and flanking deletions), and RDR3 (ΔI210 and ΔN211), we found that RDR4 (previously defined as positions 242–248) has recently expanded to include positions 249–253. These residues are indeed part of the structurally mapped supersite^13,14^, and the Lambda variant harboring the Δ246–253 deletion increased in prevalence during a test positivity surge in Chile. The ΔF157/R158 deletion (in the Delta variant), which had expanded during the massive surge in India, marked a new recurrent deletion region which also maps to the supersite^14^. Experimental studies suggest that several of these mutations are associated with increased infectivity and/or reduced neutralization by antibodies from convalescent sera (Table S6). Finally, our surveillance of clinically annotated SARS-CoV-2 genomes among COVID-19 cases at the Mayo Clinic, including vaccine breakthrough infections, revealed contiguous deletions (Δ85–90) that are distinct from the background population and are beginning to appear in other parts of United States and the world (Fig. 3). The striking trend that the most frequently deleted NTD regions are proximal to a single antigenic supersite highlights the prominent role that host immunity has played in shaping the genomic evolution of SARS-CoV-2 from the beginning of this pandemic.

There are a few limitations of this study. First, the geographic distribution of sequences deposited in the GISAID database is not representative of the global population, with a majority of the sequences coming from a small group of countries. Future genomic epidemiology studies would be improved by expanded sequencing efforts globally. Second, the identification of mutations associated with surges during early months of the pandemic is complicated by the relative paucity of whole genome sequencing data deposited during that time. Third, we have defined surge-associated mutations as those mutations which increase in prevalence monotonically in a 3-month window during which there is a monotonic increase in cases. This definition would exclude potentially surge-associated mutations if there is a delay between the emergence of mutations and their associated surge cases and if test positivity rate is decreasing temporarily due to vaccination or other reasons. Finally, the publicly accessible genomic data is generally not linked to relevant phenotypic information (e.g., disease severity) or relevant medical histories (e.g., comorbidities and vaccination status). Thus, while we can identify correlations between mutational prevalence and case surges, we cannot determine whether particular mutations are associated with more severe disease or are observed more frequently than expected by chance in vaccinated individuals. While the latter shortcoming is partially addressed by our independent whole genome sequencing of virus isolated from COVID-19 cases with accessible longitudinal records (including previously vaccinated individuals), this analysis was limited by the small size of the cohort (n = 102) and the lack of corresponding antibody titer data.

Taken together, by synthesizing insights from genomic epidemiology and clinical genomics datasets, we uncovered that SARS-CoV-2 likely employs antigenic minimalism in the Spike protein as a strategy to evade immune responses induced by infection or vaccination. These findings have important therapeutic and public health policy implications. The repertoire of deletion mutations in the N-terminal domain should be considered when developing future vaccines and biologics to counter the immuno-evasive strategies of SARS-CoV-2. From a public health standpoint, we must expand sequencing efforts around the world and encourage the transparent linking of relevant deidentified patient phenotypic data (e.g., disease severity, vaccination status) to each deposited SARS-CoV-2 genome. While the current analysis focuses on the Spike protein, analyses focusing on other SARS-CoV-2 proteins, such as the nucleocapsid protein and RNA-directed RNA polymerase, will shed further light on how the SARS-CoV-2 proteome has evolved to improve viral fitness and facilitate immune evasion. A holistic understanding of the mutational landscape of SARS-CoV-2 is imperative to proactively predict variants that could trigger outbreaks and vaccine breakthroughs, as well as to guide the development of therapeutic strategies to defeat the COVID-19 pandemic.

## Methods

### Analysis of publicly deposited SARS-CoV-2 genomic sequences

In total, 2,212,827 sequences were obtained from GISAID (as of 30 June 2021, last accessed https://www.gisaid.org/ on 5 July 2021.) For downstream analyses we ensured that all GISAID entries corresponded to the human host and had an exact collection date (YYYY-mm-dd) after 1 December 2019. This resulted in 2,128,574 SARS-CoV-2 genome sequences (with 1291 unique PANGO lineages and 11,805 unique Spike mutations) from GISAID^16^ across 188 countries/territories. To filter out potential sequencing artifacts, we excluded mutations that were present in fewer than 100 sequences, resulting in 1045 unique Spike protein mutations.

### Identification of surge-associated SARS-CoV-2 mutations

To identify mutations that have been temporally associated with surges in COVID-19 cases throughout the pandemic, we assessed monthly mutational prevalence and test positivity over 3-month intervals in each country. For each of the 1045 mutations, the monthly mutational prevalence was computed for a given country as:

$$Mutational \,Prevalence = \frac{Number \;of\; sequences \;with \;a \;mutation \;in \;a \;given\; month}{{Total\; number\; of\; sequences \;deposited \;in\; that\; month}} \times 100$$

Positivity data for PCR tests was obtained from the OWID resource^17,31^ (retrieved from https://github.com/owid/covid-19-data/tree/master/public/data on 30 June 2021). For each country, the monthly test positivity was calculated as:

$$Test \;Positivity = \frac{{New \;cases \;in\; a \;given\; month \left( {smoothened} \right)}}{{New\; tests \;in \;that \;month \left( {smoothened} \right)}} \times 100$$

To identify surge-associated mutations, we classified the monthly mutational prevalence (for each mutation) and the monthly test positivity as increasing (monotonically), decreasing (monotonically), or mixed over sliding 3-month intervals over the course of the pandemic. Any mutation which monotonically increased in prevalence over this interval in a country with a simultaneous monotonic increase in test positivity was defined as a “surge-associated mutation.” There were 89 such mutations.

### Comparison of surge-associated mutations to mutations in CDC variants of interest and concern

In order to test the value of our method, we obtained the set of CDC variants of interest and concern as of 13 July 13 2021^8^. At this time, there were 4 variants of concern (Alpha_B.1.1.7, Beta_B.1.351, Delta_B.1.617.2 and Gamma_P.1) and 7 variants of interest (Epsilon_B.1.427, Epsilon_B.1.425, Eta_B.1.525, Iota_B.1.1526, Kappa_B.1.617.1, B.1.617.3, Zeta_P.2), with no variants of high consequence. From the 11 classified variants, there are 59 unique mutations (53 positions), of which 18 were found only in variants of interest, 29 were found only in variants of concern, and 12 were found in both variants of interest and concern. After identifying the surge-associated mutations as described above, we determined the fraction of mutations comprising the CDC-classified variants which were captured by this approach.

### Assessment of mutation types for enrichment of surge-associated mutations

After identifying the 92 surge-associated mutations, we tested whether any of the contributing mutation types (deletions, insertions, or substitutions) were enriched for surge-associated mutations. To do so, we constructed a 2 × 3 table giving the number of surge-associated and non-surge-associated mutations in each category. To determine whether one or more groups showed a statistically significant enrichment, a chi-square *p* value was calculated using the scipy.stats.chi2_contingency function from the scipy package (1.7.0) in Python v3.9.5. Post-hoc Fisher's tests were performed by constructing 2 × 2 contingency tables to compare each mutation type against all others. Then, odds ratios and their corresponding 95% confidence intervals were calculated using the scipy.stats.fisher_exact function and statsmodels.stats.contingency_tables.Table2 × 2 respectively in Python v3.9.5.

### Identification of recurrent deletion regions in the Spike protein

Recurrent deletion regions (RDRs) were previously defined as four sites within the NTD within which over 90% of all Spike protein deletions occurred, per the 146,795 SARS-CoV-2 sequences deposited in GISAID from 1 December 2019 to 24 October 2020^15^.

To formally identify RDRs that have emerged over the course of the pandemic, we considered the monthly distribution of deletion counts for each amino acid (i.e. number of sequences in which deletion of the given amino acid was observed in a given month) in the Spike protein. For each month, we calculated the 95th percentile of the deletion count distribution. We then bucketed each residue *R* into categories (Yes, No, Possible) reflecting whether or not it should be considered as part of an RDR (i.e., a contiguous stretch of two or more amino acid residues which undergo deletion events more frequently than expected by chance) for that month as follows (illustrated schematically in Table S2).

Once each residue was categorized in this way, then any residue *P* in the “Possible” category were subjected to further analysis to convert their labels into “Yes” or “No.” Specifically, we took a stepwise approach, walking in both directions from *P* until the first encounter of a residue categorized as “Yes” or “No” (i.e., other residues labeled as “Possible” were ignored). If a residue categorized as “Yes” was encountered before any residue categorized as “No” in either direction, then the “Possible” label was converted to “Yes.” If a residue categorized as “No” was encountered before any residue categorized as “Yes” in both directions, then the “Possible” label was converted to “No”. With each residue categorized as “Yes” or “No”, we then simply merged the residue windows with consecutive “Yes” labels to define the updated set of Spike protein RDRs for that month.

### Temporal analysis of expansions in recurrent deletion regions

To assess the expansion of regions undergoing deletions over time, we plotted a time series tile plot indicating each month in which a given deletion was identified as part of an RDR (based on all GISAID sequences deposited in that month). The residues plotted were defined based on the definition of RDRs provided above, which builds upon the regions defined previously^15^. Amino acids which were included in the previously defined RDRs were indicated in the plot to distinguish them from amino acids which (1) are part of newly emerged RDRs or (2) represent contiguous expansions from a previously defined RDR.

### Structural analysis of SARS-CoV-2 Spike protein

Structural analyses and illustrations were performed in PyMOL (version 2.3.4). The cryo-EM structure of the Spike protein characterizing the interaction with a neutralizing antibody 4A8 (PDB identifier: 7C2L), described by Chi et al.^18^, was retrieved from the PDB.

### Amplicon sequencing of SARS-CoV-2 genome obtained from individuals with breakthrough infections

This is a retrospective study of individuals who underwent polymerase chain reaction (PCR) testing for suspected SARS-CoV-2 infection at the Mayo Clinic and hospitals affiliated to the Mayo health system.

SARS-CoV-2 RNA-positive upper respiratory tract swab specimens from patients with vaccine breakthrough or reinfection of COVID-19 were subjected to next-generation sequencing, using the commercially available Ion AmpliSeq SARS-CoV-2 Research Panel (Life Technologies Corp., South San Francisco, CA) based on the "sequencing by synthesis" method. The assay amplifies 237 sequences ranging from 125 to 275 base pairs in length, covering 99% of the SARS-CoV-2 genome. Viral RNA was first manually extracted and purified from these clinical specimens using MagMAX™ Viral/Pathogen Nucleic Acid Isolation Kit (Life Technologies Corp.), followed by automated reverse transcription-PCR (RT-PCR) of viral sequences, DNA library preparation (including enzymatic shearing, adapter ligation, purification, normalization), DNA template preparation, and sequencing on the automated Genexus™ Integrated Sequencer (Life Technologies Corp.) with the Genexus™ Software version 6.2.1. A no-template control and a positive SARS-CoV-2 control were included in each assay run for quality control purposes. Viral sequence data were assembled using the Iterative Refinement Meta-Assembler (IRMA) application (50% base substitution frequency threshold) to generate unamended plurality consensus sequences for analysis with the latest versions of the web-based application tools: Pangolin^32^ for SARS-CoV-2 lineage assignment; Nextclade^33^ for viral clade assignment, phylogenetic analysis, and S codon mutation calling, in comparison to the wild-type reference sequence of SARS-CoV-2 Wuhan-Hu-1 (lineage B, clade 19A).

The SARS-CoV-2 sequences have been made available through the GISAID database (https://gisaid.org/). The database identifiers are as follows: EPI_ISL_12916271, EPI_ISL_12916270, EPI_ISL_12916273, EPI_ISL_12916272, EPI_ISL_12916275, EPI_ISL_12916310, EPI_ISL_12916274, EPI_ISL_12916277, EPI_ISL_12916276, EPI_ISL_12916313, EPI_ISL_12916279, EPI_ISL_12916314, EPI_ISL_12916278, EPI_ISL_12916311, EPI_ISL_12916312, EPI_ISL_12916317, EPI_ISL_12916318, EPI_ISL_12916315, EPI_ISL_12916316, EPI_ISL_12916319, EPI_ISL_12916260, EPI_ISL_12916262, EPI_ISL_12916261, EPI_ISL_12916264, EPI_ISL_12916263, EPI_ISL_12916266, EPI_ISL_12916265, EPI_ISL_12916302, EPI_ISL_12916268, EPI_ISL_12916303, EPI_ISL_12916267, EPI_ISL_12916300, EPI_ISL_12916301, EPI_ISL_12916269, EPI_ISL_12916306, EPI_ISL_12916307, EPI_ISL_12916304, EPI_ISL_12916305, EPI_ISL_12916308, EPI_ISL_12916309, EPI_ISL_12916251, EPI_ISL_12916250, EPI_ISL_12916253, EPI_ISL_12916252, EPI_ISL_12916255, EPI_ISL_12916254, EPI_ISL_12916257, EPI_ISL_12916256, EPI_ISL_12916259, EPI_ISL_12916258, EPI_ISL_12916240, EPI_ISL_12916242, EPI_ISL_12916241, EPI_ISL_12916244, EPI_ISL_12916243, EPI_ISL_12916246, EPI_ISL_12916245, EPI_ISL_12916248, EPI_ISL_12916247, EPI_ISL_12916249, EPI_ISL_12916239, EPI_ISL_12916238, EPI_ISL_12916290, EPI_ISL_12916291, EPI_ISL_12916294, EPI_ISL_12916295, EPI_ISL_12916292, EPI_ISL_12916293, EPI_ISL_12916298, EPI_ISL_12916331, EPI_ISL_12916332, EPI_ISL_12916299, EPI_ISL_12916296, EPI_ISL_12916297, EPI_ISL_12916330, EPI_ISL_12916335, EPI_ISL_12916336, EPI_ISL_12916333, EPI_ISL_12916334, EPI_ISL_12916339, EPI_ISL_12916337, EPI_ISL_12916338, EPI_ISL_12916280, EPI_ISL_12916283, EPI_ISL_12916284, EPI_ISL_12916281, EPI_ISL_12916282, EPI_ISL_12916320, EPI_ISL_12916287, EPI_ISL_12916321, EPI_ISL_12916288, EPI_ISL_12916285, EPI_ISL_12916286, EPI_ISL_12916324, EPI_ISL_12916325, EPI_ISL_12916289, EPI_ISL_12916322, EPI_ISL_12916323, EPI_ISL_12916328, EPI_ISL_12916329, EPI_ISL_12916326, EPI_ISL_12916327.

### Ethical approval

This work was reviewed by the Mayo Clinic IRB and determined to be exempt from human subjects research as it is a secondary use of data which was de-identified for analysis (45 CFR 46.104d, category 4).

## Supplementary Information

Supplementary Information.

## Data Availability

After publication, the data will be made available upon reasonable requests to the corresponding author. A proposal with a detailed description of study objectives and the statistical analysis plan will be needed for evaluation of the reasonability of requests. These SARS-CoV-2 genome sequences have been made available through the GISAID database. The accession numbers are as follows: EPI_ISL_12916271, EPI_ISL_12916270, EPI_ISL_12916273, EPI_ISL_12916272, EPI_ISL_12916275, EPI_ISL_12916310, EPI_ISL_12916274, EPI_ISL_12916277, EPI_ISL_12916276, EPI_ISL_12916313, EPI_ISL_12916279, EPI_ISL_12916314, EPI_ISL_12916278, EPI_ISL_12916311, EPI_ISL_12916312, EPI_ISL_12916317, EPI_ISL_12916318, EPI_ISL_12916315, EPI_ISL_12916316, EPI_ISL_12916319, EPI_ISL_12916260, EPI_ISL_12916262, EPI_ISL_12916261, EPI_ISL_12916264, EPI_ISL_12916263, EPI_ISL_12916266, EPI_ISL_12916265, EPI_ISL_12916302, EPI_ISL_12916268, EPI_ISL_12916303, EPI_ISL_12916267, EPI_ISL_12916300, EPI_ISL_12916301, EPI_ISL_12916269, EPI_ISL_12916306, EPI_ISL_12916307, EPI_ISL_12916304, EPI_ISL_12916305, EPI_ISL_12916308, EPI_ISL_12916309, EPI_ISL_12916251, EPI_ISL_12916250, EPI_ISL_12916253, EPI_ISL_12916252, EPI_ISL_12916255, EPI_ISL_12916254, EPI_ISL_12916257, EPI_ISL_12916256, EPI_ISL_12916259, EPI_ISL_12916258, EPI_ISL_12916240, EPI_ISL_12916242, EPI_ISL_12916241, EPI_ISL_12916244, EPI_ISL_12916243, EPI_ISL_12916246, EPI_ISL_12916245, EPI_ISL_12916248, EPI_ISL_12916247, EPI_ISL_12916249, EPI_ISL_12916239, EPI_ISL_12916238, EPI_ISL_12916290, EPI_ISL_12916291, EPI_ISL_12916294, EPI_ISL_12916295, EPI_ISL_12916292, EPI_ISL_12916293, EPI_ISL_12916298, EPI_ISL_12916331, EPI_ISL_12916332, EPI_ISL_12916299, EPI_ISL_12916296, EPI_ISL_12916297, EPI_ISL_12916330, EPI_ISL_12916335, EPI_ISL_12916336, EPI_ISL_12916333, EPI_ISL_12916334, EPI_ISL_12916339, EPI_ISL_12916337, EPI_ISL_12916338, EPI_ISL_12916280, EPI_ISL_12916283, EPI_ISL_12916284, EPI_ISL_12916281, EPI_ISL_12916282, EPI_ISL_12916320, EPI_ISL_12916287, EPI_ISL_12916321, EPI_ISL_12916288, EPI_ISL_12916285, EPI_ISL_12916286, EPI_ISL_12916324, EPI_ISL_12916325, EPI_ISL_12916289, EPI_ISL_12916322, EPI_ISL_12916323, EPI_ISL_12916328, EPI_ISL_12916329, EPI_ISL_12916326, EPI_ISL_12916327.
