# Supplementary material for: Expanding repertoire of SARS-CoV-2 deletion mutations contributes to evolution of highly transmissible variants
Source: Sci Rep. 2023 Jan 5;13:257. doi: 10.1038/s41598-022-26646-5 (PMC9815892; doi:10.1038/s41598-022-26646-5)
Supplement: Supplementary file 1 — Supplementary Information. [file 41598_2022_26646_MOESM1_ESM.docx]

**Supplementary Information**

**Expanding repertoire of SARS-CoV-2 deletion mutations contributes to evolution of highly transmissible variants**

A.J. Venkatakrishnan^1+^*, Praveen Anand^2+^, Patrick Lenehan^1^, Pritha Ghosh^2^, Rohit Suratekar^2^, Eli Silvert^1^, Colin Pawlowski^1^, Abhishek Siroha^2^, Dibyendu Roy Chowdhury^1^, John C. O’Horo^3^, Joseph D. Yao^3^, Bobbi S. Pritt^3^, Andrew Norgan^3^, Ryan T. Hurt^3^, Andrew D. Badley^3^, John Halamka^3^, Venky Soundararajan^1,2,4^*

^1^ nference, Cambridge, Massachusetts 02139, USA

^2^ nference Labs, Bengaluru, Karnataka, India

^3^Mayo Clinic, Rochester, Minnesota 55902, USA

^4^Anumana, Cambridge, Massachusetts 02139, USA

- Equal first authors

* Correspondence to: Venky Soundararajan ([venky@nference.net](mailto:venky@nference.net)), A.J. Venkatakrishnan ([aj@nference.net](mailto:aj@nference.net))

**
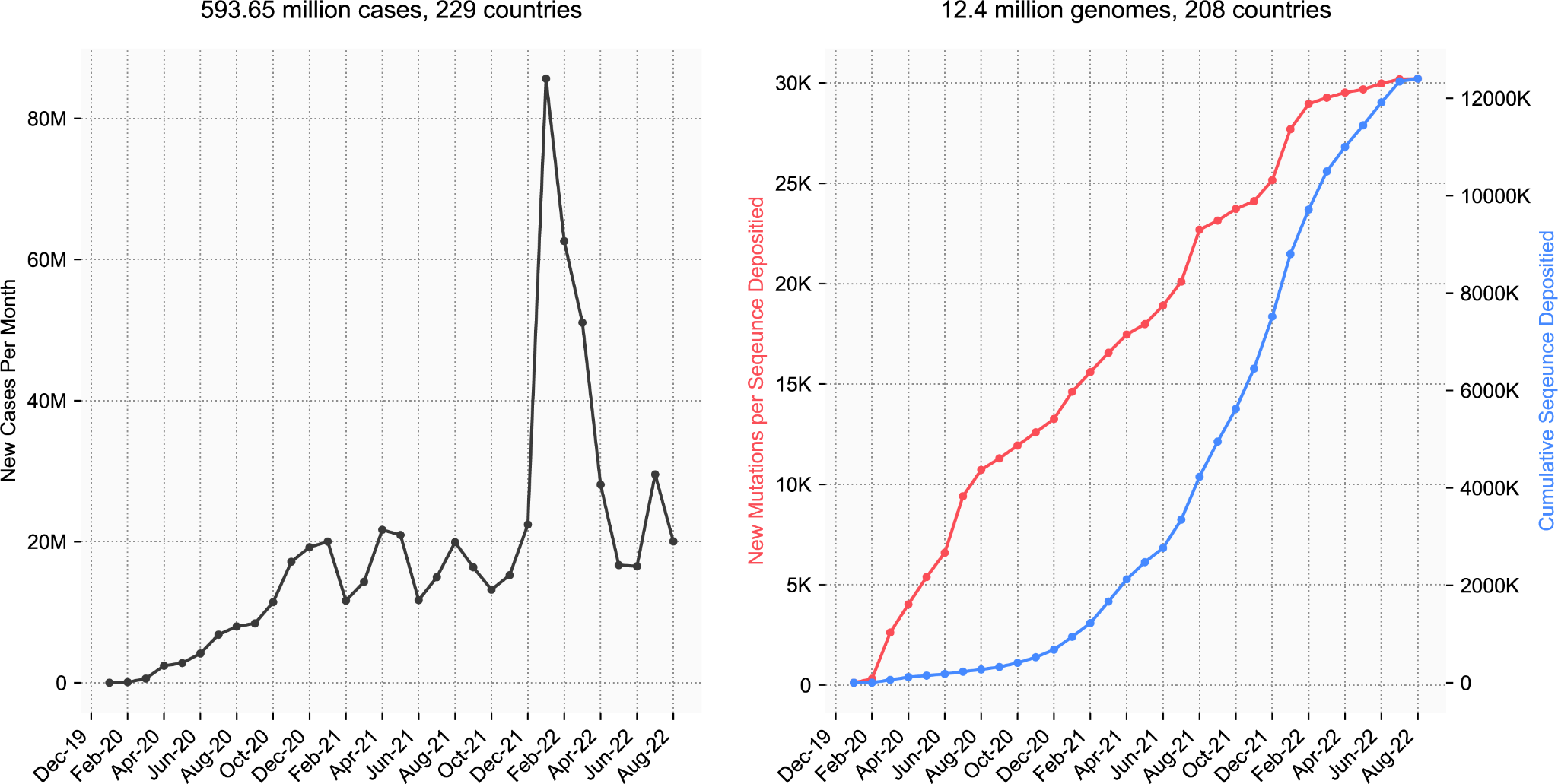
**

**Figure S1. Overview of COVID-19 incidence and accumulation of SARS-CoV-2 mutations globally during the pandemic** (updated as of 22 August 2022).

**
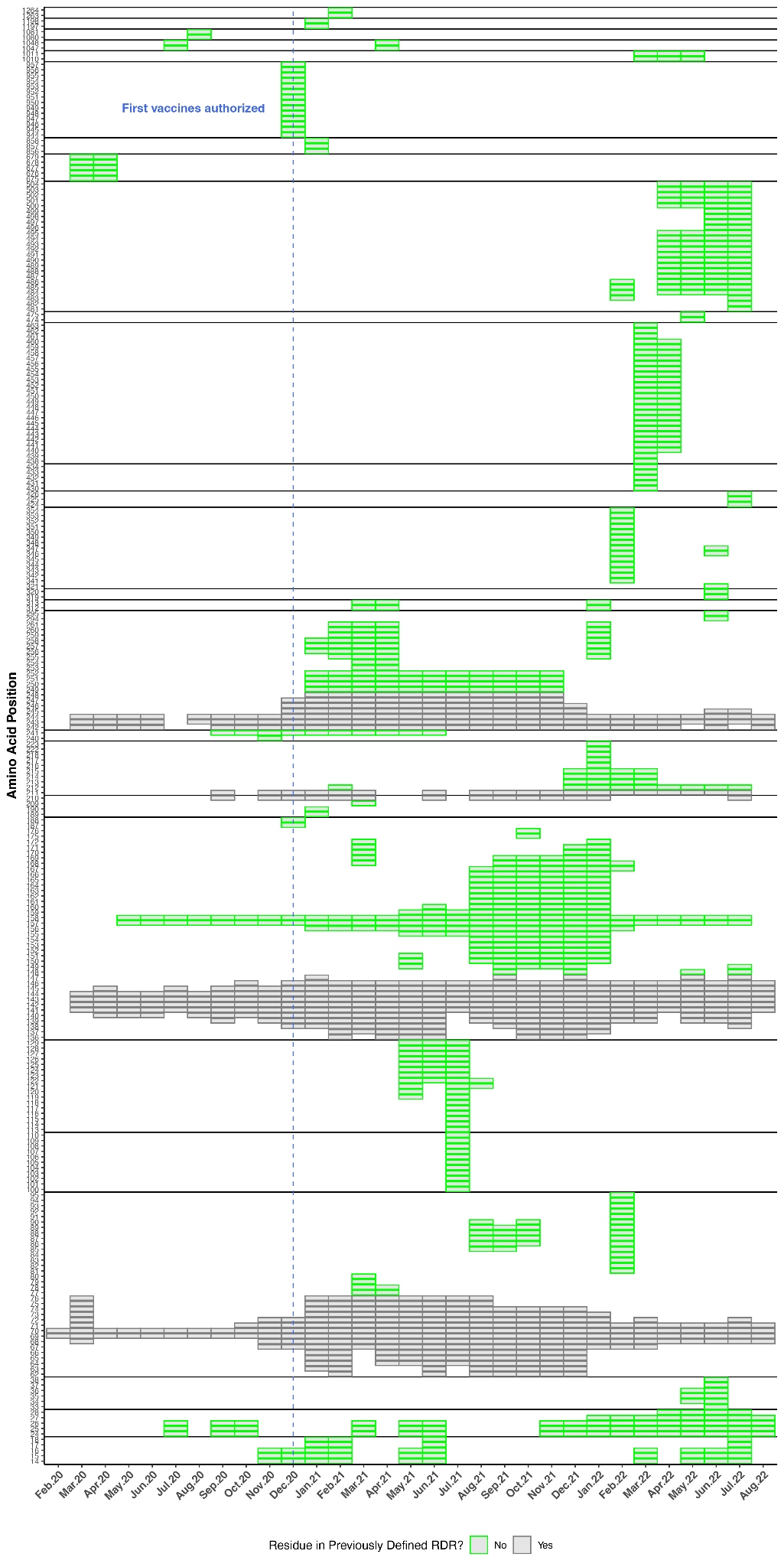
**

**Figure S2. The repertoire of deletions in the Spike protein N-terminal domain is expanding over the course of the pandemic.** Heatmap showing the expansion of “deletable” regions in the course of the pandemic (updated as of 22 August 2022), where the rows denote residue positions in the Spike protein and columns denote the time course of the pandemic (in months). The boxes denote the frequency of the deletion mutation across the world in that month.

**
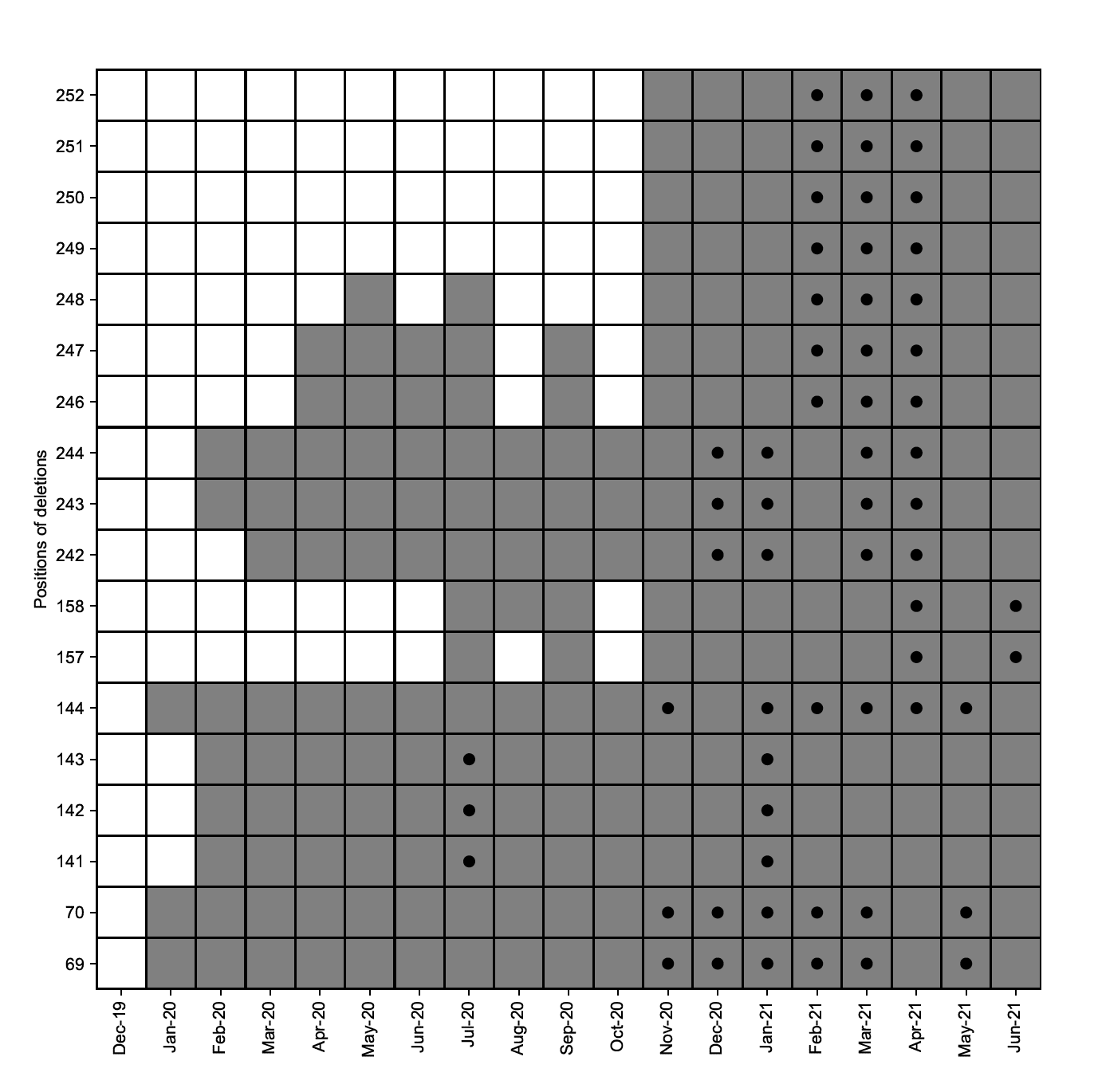
**

**Figure S2: Occurrence of surge-associated deletions in the course of the pandemic.** Cells in grey indicate months in which a particular deletion mutation is observed in one or more sequences. The cells marked with black dots indicate months in which these mutations are associated with surges in community transmission.


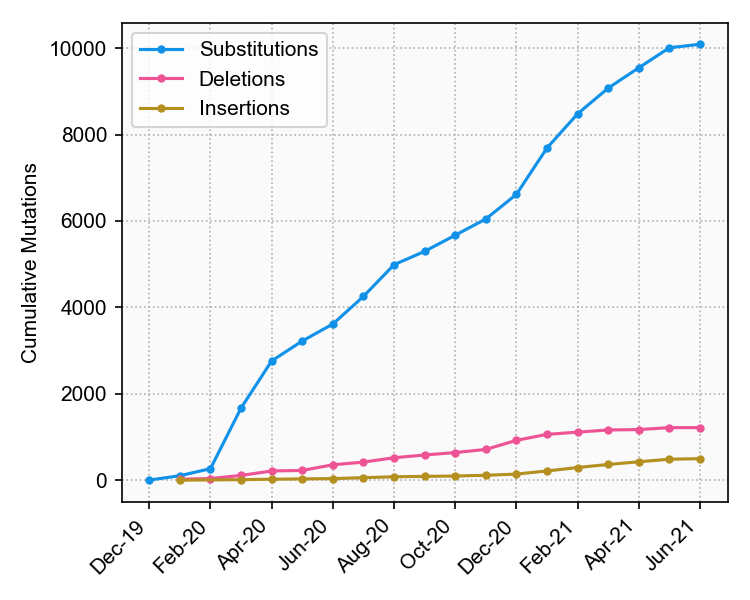


**Figure S4:** Accumulation of substitutions, insertions, and deletions in the SARS-CoV-2 Spike protein over the course of the pandemic. Deletions have increased more compared to insertions. As shown by other studies for the early phase of the pandemic, substitutions have remained as the dominant mutational event. However, the following should be considered while interpreting the mutational preference: (i) Sequencing efforts were significantly increased in the later stages of the pandemic, and hence mutation events happening very early in the pandemic might have been missed, and (ii) The mutations identified are heavily biased by those occurring in high sequencing countries, like the United States and the United Kingdom.

**Table S1:** List of all 27 deletions in the N-terminal domain that were identified to be either associated or not associated with surge (please see Figure 1c). The number of GISAID entries (as of 30 June 2021) where the deletions were detected along with the total number of countries the deletion mutation was observed is tabulated here.

| **Spike Deletion** | **Surge Associated** | **No. of GISAID Sequences** | **No. of Countries detected** |
| --- | --- | --- | --- |
| ΔH69 | Yes | 944987 | 155 |
| ΔV70 | Yes | 946205 | 155 |
| ΔL141 | Yes | 2820 | 79 |
| ΔG142 | Yes | 3807 | 80 |
| ΔV143 | Yes | 3956 | 79 |
| ΔY144 | Yes | 931161 | 160 |
| ΔF157 | Yes | 98367 | 78 |
| ΔR158 | Yes | 98334 | 78 |
| ΔL242 | Yes | 24867 | 102 |
| ΔA243 | Yes | 25792 | 105 |
| ΔL244 | Yes | 25302 | 104 |
| ΔR246 | Yes | 2058 | 40 |
| ΔS247 | Yes | 1938 | 37 |
| ΔY248 | Yes | 1870 | 31 |
| ΔL249 | Yes | 1837 | 25 |
| ΔT250 | Yes | 1838 | 25 |
| ΔP251 | Yes | 1840 | 25 |
| ΔG252 | Yes | 1841 | 25 |
| ΔS71 | No | 1703 | 45 |
| ΔD138 | No | 320 | 28 |
| ΔP139 | No | 389 | 32 |
| ΔF140 | No | 679 | 51 |
| ΔY145 | No | 729 | 40 |
| ΔH146 | No | 963 | 39 |
| ΔI210 | No | 989 | 38 |
| ΔN211 | No | 187 | 25 |
| ΔL241 | No | 252 | 29 |

**Table S2:** Schematic representation of the decision schema for considering a residue *R* to be a part of a RDR. Deletion count of below the 95th percentile for a given month is represented by **X.** Deletion count above the 95th percentile for a given month is represented by ✔.

| Deletion count | | | | | Should Residue *R* be considered part of a RDR in the given month? |
| --- | --- | --- | --- | --- | --- |
| *R* - 2 | *R* - 1 | Residue *R* | *R* + 1 | *R* + 2 |  |
|  | X | X |  |  | No |
|  |  | X | X |  | No |
|  | ✔ | ✔ |  |  | Yes |
|  |  | ✔ | ✔ |  | Yes |
|  | ✔ |  | ✔ | ✔ | Yes |
| ✔ | ✔ |  | ✔ |  | Yes |
| X | X | ✔ |  | ✔ | Possible |
| ✔ |  | ✔ | X | X | Possible |
|  | ✔ | X | ✔ |  | Possible |

**Table S3:** In total, 102 COVID-19 patients from Mayo clinic had SARS-CoV-2 genome profiled from their swab specimen samples. The inclusion criteria for sequencing of SARS-CoV-2 genome along with the demographic information such as age, gender, race and ethnicity for all the 102 patients has been tabulated here. The table also indicates the PANGO lineage and the Nextstrain clade assignment along with the variants identified from each of the assembled SARS-CoV-2 genome from individual patients. The information on type of SARS-CoV-2 vaccine received by individual patients where available is also tabulated here along with dose information (*single dose/ ** complete dose).

| **Patient** | **Gender** | **Ethnicity** | **PANGO lineage** | **Nextstrain clade** | **Mutations** | **Inclusion criteria** | **Vaccine Type** | **Vaccine DOSE** |
| --- | --- | --- | --- | --- | --- | --- | --- | --- |
| Patient1 | M | Not Hispanic or Latino | B.1.1.7 | 20I | N501Y, A570D, D614G, P681H, T716I, S982A, D1118H, K1191N, H69-V70 deletion, Y144 deletion | Post-vaccine infection | SARS-COV-2 (COVID-19) - PFIZER | ** |
| Patient2 | M | Not Hispanic or Latino | B.1.1.7 | 20I | N501Y, A570D, D614G, P681H, T716I, H69-V70 deletion, Y144 deletion, V126F, S982A, D1118H | Post-vaccine infection | SARS-COV-2 (COVID-19) - PFIZER | ** |
| Patient3 | F | Not Hispanic or Latino | B.1.1.7 | 20I | N501Y, A570D, D614G, P681H, T716I, S982A, D1118H, H69-V70 deletion, Y144 deletion | Post-vaccine infection | SARS-COV-2 (COVID-19) - PFIZER | ** |
| Patient4 | F | Not Hispanic or Latino | B.1.1.7 | 20I | N501Y, A570D, D614G, P681H, T716I, H69-V70 deletion, Y144 deletion, L18F, S982A, D1118H | Post-vaccine infection | SARS-COV-2 (COVID-19) - PFIZER | ** |
| Patient5 | F | Not Hispanic or Latino | B.1.429 | 20C | L452R, D614G, Q173-, S13I, F86X, W152C, C1043X, T167-, F168-, E169-, Y170-, V171-, S172-, P174- | Post-vaccine infection | NA | NA |
| Patient6 | F | Not Hispanic or Latino | B.1.1.7 | 20I | N501Y, A570D, D614G, P681H, T716I, H69-V70 deletion, Y144 deletion, S982A, D1118H | Hospitalized for COVID-19 | NA | NA |
| Patient7 | M | Not Hispanic or Latino | B.1.1.7 | 20I | N501Y, A570D, D614G, P681H, T716I, H69-V70 deletion, Y144 deletion | Post-vaccine infection | SARS-COV-2 (COVID-19) - JANSSEN (J&J) | ** |
| Patient8 | M | Not Hispanic or Latino | B.1.1.7 | 20I | N501Y, A570D, D614G, P681H, T716I, H69-V70 deletion, Y144 deletion, P85Q, N87X, Y91X, S982A, D1118H, K1191N, D88- | Post-vaccine infection | SARS-COV-2 (COVID-19) - JANSSEN (J&J) | ** |
| Patient9 | M | Not Hispanic or Latino | B.1.1.7 | 20I | F490S, N501Y, A570D, D614G, P681H, T716I, H69-V70 deletion, Y144 deletion, F86X, D138H, S982A, D1118H, C1235F | Hospitalized for COVID-19 | NA | NA |
| Patient10 | M | Not Hispanic or Latino | B.1.351 | 20H | L18F, D80A, D215G, L242H, K417N, E484K, N501Y, D614G, A701V, P812L, A243-, L244-, H245- | Post-vaccine infection | SARS-COV-2 (COVID-19) - PFIZER | ** |
| Patient11 | F | Not Hispanic or Latino | B.1.1.7 | 20I | N501Y, A570D, D614G, P681H, T716I, H69-V70 deletion, Y144 deletion, F86X, S982A, D1118H | Post-vaccine infection | NA | NA |
| Patient12 | NA | NA | B.1.1.7 | 20I | N501Y, A570D, D614G, P681H, T716I, H69-V70 deletion, Y144 deletion | Post-vaccine infection | NA | NA |
| Patient13 | M | Not Hispanic or Latino | B.1.1.7 | 20I | N501Y, A570D, D614G, P681H, T716I, H69-V70 deletion, Y144 deletion, S982A, D1118H, K1191N | Post-vaccine infection | SARS-COV-2 (COVID-19) - PFIZER | ** |
| Patient14 | F | Not Hispanic or Latino | B.1.1.7 | 20I | N501Y, A570D, D614G, P681H, T716I, S982A, D1118H, H69-V70 deletion, Y144 deletion | Post-vaccine infection | SARS-COV-2 (COVID-19) - PFIZER | ** |
| Patient15 | NA | NA | B.1.1.519 | 20B | D614G, P681H | Hospitalized for COVID-19 | NA | NA |
| Patient16 | F | Not Hispanic or Latino | B.1.1.7 | 20I | P85L, G89V, N501Y, A570D, D614G, P681H, T716I, S982A, D1118H, K1191N, H69-V70 deletion, F86-, V90-, Y144 deletion | Hospitalized for COVID-19 | NA | None |
| Patient17 | F | Not Hispanic or Latino | B.1.1.7 | 20I | N501Y, A570D, D614G, P681H, T716I, H69-V70 deletion, Y144 deletion, S982A, D1118H, K1191N | Post-vaccine infection | SARS-COV-2 (COVID-19) - JANSSEN (J&J) | ** |
| Patient18 | F | Not Hispanic or Latino | B.1.1.7 | 20I | N501Y, A570D, D614G, P681H, T716I, H69-V70 deletion, Y144 deletion, S982A, D1118H, K1191N | Post-vaccine infection | SARS-COV-2 (COVID-19) - PFIZER | ** |
| Patient19 | NA | NA | B.1.1.7 | 20I | N501Y, A570D, D614G, P681H, T716I, H69-V70 deletion, Y144 deletion | Post-vaccine infection | SARS-COV-2 (COVID-19) - PFIZER | ** |
| Patient20 | M | Not Hispanic or Latino | B.1.1.7 | 20I | N501Y, A570D, D614G, P681H, T716I, H69-V70 deletion, Y144 deletion, L118F, S982A, D1118H, V1268I | Post-vaccine infection | NA | NA |
| Patient21 | F | Not Hispanic or Latino | B.1.1.519 | 20B | D614G, P681H, T478K, E516Q, T732A | Hospitalized for COVID-19 | NA | None |
| Patient22 | F | Not Hispanic or Latino | B.1.1.7 | 20I | L118F, N501Y, A570D, D614G, P681H, T716I, S982A, D1118H, H69-V70 deletion, Y144 deletion | Post-vaccine infection | NA | NA |
| Patient23 | F | Not Hispanic or Latino | B.1.1.7 | 20I | N501Y, A570D, D614G, P681H, T716I, H69-V70 deletion, Y144 deletion, S704L, S982A, D1118H | Post-vaccine infection | SARS-COV-2 (COVID-19) - JANSSEN (J&J) | ** |
| Patient24 | F | Not Hispanic or Latino | B.1.1.7 | 20I | N501Y, A570D, D614G, P681H, T716I, H69-V70 deletion, Y144 deletion, S982A, D1118H | Post-vaccine infection | SARS-COV-2 (COVID-19) - PFIZER | ** |
| Patient25 | F | Not Hispanic or Latino | B.1.1.7 | 20I | F86X, N501Y, A570D, D614G, P681H, S704L, T716I, S982A, D1118H, H69-V70 deletion, Y144 deletion | Post-vaccine infection | SARS-COV-2 (COVID-19) - PFIZER | * |
| Patient26 | F | Not Hispanic or Latino | B.1.1.7 | 20I | N501Y, A570D, D614G, P681H, T716I, H69-V70 deletion, Y144 deletion, F86X, M153I, S982A, D1118H, K1191N, V90- | Hospitalized for COVID-19 | SARS-COV-2 (COVID-19) - MODERNA | * |
| Patient27 | NA | NA | B.1.526 | 20C | D253G, E484K, D614G, A701V, L5X, T95I, L141X, N148X, A845S | Post-vaccine infection | NA | NA |
| Patient28 | F | Not Hispanic or Latino | B.1.429 | 20C | L452R, D614G, S13I, W152C, E156-, F157-, R158-, V159-, Y160-, S161-, S162-, A163-, N164- | Post-vaccine infection | SARS-COV-2 (COVID-19) - PFIZER | ** |
| Patient29 | M | Not Hispanic or Latino | B.1.1.7 | 20I | N501Y, A570D, D614G, P681H, T716I, H69-V70 deletion, Y144 deletion, F86X, S982A, D1118H, K1191N | Post-vaccine infection | NA | NA |
| Patient30 | F | Not Hispanic or Latino | B.1.1.7 | 20I | N501Y, A570D, D614G, P681H, T716I, H69-V70 deletion, Y144 deletion, S982A, D1118H, K1191N | Hospitalized for COVID-19 | SARS-COV-2 (COVID-19) - PFIZER | * |
| Patient31 | F | Not Hispanic or Latino | B.1.1.7 | 20I | N501Y, A570D, D614G, P681H, T716I, H69-V70 deletion, Y144 deletion, S982A, D1118H, K1191N | Post-vaccine infection | SARS-COV-2 (COVID-19) - MODERNA | ** |
| Patient32 | F | Not Hispanic or Latino | B.1.526.1 | 20C | L452R, D614G, Y144 deletion, D80G, F157S, T859N, D950H, D1257E | Post-vaccine infection | SARS-COV-2 (COVID-19) - PFIZER | ** |
| Patient33 | NA | NA | B.1.1.7 | 20I | H49Y, N501Y, A570D, D614G, P681H, T716I, S982A, D1118H, H69-V70 deletion, Y144 deletion | Post-vaccine infection | SARS-COV-2 (COVID-19) - JANSSEN (J&J) | ** |
| Patient34 | F | Not Hispanic or Latino | B.1.1.7 | 20I | N501Y, A570D, D614G, P681H, T716I, H69-V70 deletion, Y144 deletion, S982A, D1118H | Post-vaccine infection | SARS-COV-2 (COVID-19) - PFIZER | ** |
| Patient35 | NA | NA | B.1.1.7 | 20I | N501Y, A570D, D614G, P681H, T716I, H69-V70 deletion, Y144 deletion | Hospitalized for COVID-19 | NA | NA |
| Patient36 | F | Not Hispanic or Latino | B.1.1.7 | 20I | N501Y, A570D, D614G, P681H, A701S, T716I, H69-V70 deletion, Y144 deletion, S982A, D1118H | Post-vaccine infection | SARS-COV-2 (COVID-19) - PFIZER | ** |
| Patient37 | F | Not Hispanic or Latino | B.1.1.7 | 20I | N501Y, A570D, D614G, P681H, T716I, H69-V70 deletion, Y144 deletion | Post-vaccine infection | SARS-COV-2 (COVID-19) - PFIZER | ** |
| Patient38 | F | Not Hispanic or Latino | B.1.1.7 | 20I | N501Y, A570D, D614G, P681H, T716I, H69-V70 deletion, Y144 deletion, S982A, D1118H | Post-vaccine infection | SARS-COV-2 (COVID-19) - PFIZER | ** |
| Patient39 | NA | NA | B.1.1.7 | 20I | N501Y, A570D, D614G, P681H, T716I, H69-V70 deletion, Y144 deletion, S982A, D1118H | Post-vaccine infection | NA | NA |
| Patient40 | F | Not Hispanic or Latino | B.1.2 | 20G | D614G, T1273I | Re-infection vs presistent infection | SARS-COV-2 (COVID-19) - PFIZER | ** |
| Patient41 | M | Not Hispanic or Latino | B.1.429 | 20C | L452R, D614G, S13I, F86X, W152C, D867G | Hospitalized for COVID-19 | SARS-COV-2 (COVID-19) - PFIZER | ** |
| Patient42 | M | Not Hispanic or Latino | B.1.429 | 20C | L452R, D614G, S13I, W152C | Hospitalized for COVID-19 | NA | None |
| Patient43 | F | Not Hispanic or Latino | B.1.1.7 | 20I | F86X, N501Y, A570D, D614G, P681H, T716I, S982A, D1118H, H69-V70 deletion, Y144 deletion | Post-vaccine infection | SARS-COV-2 (COVID-19) - PFIZER | ** |
| Patient44 | F | Not Hispanic or Latino | B.1.1.7 | 20I | N501Y, A570D, D614G, P681H, T716I, H69-V70 deletion, Y144 deletion, S982A, D1118H, K1191N | Post-vaccine infection | NA | NA |
| Patient45 | F | Not Hispanic or Latino | B.1.1.7 | 20I | N501Y, A570D, D614G, P681H, S704L, T716I, S982A, D1118H, H69-V70 deletion, Y144 deletion | Hospitalized for COVID-19 | NA | None |
| Patient46 | NA | NA | B.1.429 | 20C | S13I, W152C, L452R, D614G | Post-vaccine infection | SARS-COV-2 (COVID-19) - PFIZER | ** |
| Patient47 | F | Not Hispanic or Latino | B.1.1.7 | 20I | N501Y, A570D, D614G, P681H, T716I, H69-V70 deletion, Y144 deletion, F86X, S982A, D1118H, K1191N | Re-infection vs presistent infection | NA | None |
| Patient48 | F | Not Hispanic or Latino | B.1.1.7 | 20I | N501Y, A570D, D614G, P681H, T716I, H69-V70 deletion, Y144 deletion, F86X, S982A, D1118H | Hospitalized for COVID-19 | NA | None |
| Patient49 | F | Not Hispanic or Latino | B.1.1.7 | 20I | N501Y, A570D, D614G, P681H, T716I, H69-V70 deletion, Y144 deletion, S982A, D1118H, K1191N | Post-vaccine infection | SARS-COV-2 (COVID-19) - PFIZER | ** |
| Patient50 | F | Not Hispanic or Latino | B.1.1.7 | 20I | N501Y, A570D, D614G, P681H, T716I, H69-V70 deletion, Y144 deletion | Post-vaccine infection | SARS-COV-2 (COVID-19) - PFIZER | ** |
| Patient51 | M | Not Hispanic or Latino | B.1.1.7 | 20I | N501Y, A570D, D614G, P681H, T716I, H69-V70 deletion, Y144 deletion, S982A, D1118H | Post-vaccine infection | SARS-COV-2 (COVID-19) - PFIZER | ** |
| Patient52 | M | Not Hispanic or Latino | B.1.1.7 | 20I | N501Y, A570D, D614G, P681H, T716I, H69-V70 deletion, Y144 deletion | Post-vaccine infection | SARS-COV-2 (COVID-19) - MODERNA | * |
| Patient53 | F | Not Hispanic or Latino | B.1.525 | 20A | E484K, D614G, Q677H, H69-V70 deletion, Y144 deletion, Q52R, A67V, F888L | Hospitalized for COVID-19 | NA | None |
| Patient54 | M | Not Hispanic or Latino | B.1.427 | 20C | L452R, D614G, S13I, W152C, T676I | Hospitalized for COVID-19 |  | None |
| Patient55 | F | Not Hispanic or Latino | B.1.429 | 20C | L452R, D614G, S13I, W152C | Post-vaccine infection | SARS-COV-2 (COVID-19) - PFIZER | ** |
| Patient56 | M | Other Spanish culture of origin regardless of race (except Spain) | B.1.1.7 | 20I | N501Y, A570D, D614G, P681H, T716I, H69-V70 deletion, Y144 deletion, W258L, S982A, D1118H | Post-vaccine infection | SARS-COV-2 (COVID-19) - PFIZER | ** |
| Patient57 | F | Not Hispanic or Latino | B.1.1.7 | 20I | N501Y, A570D, D614G, P681H, T716I, H69-V70 deletion, Y144 deletion, S704L, S982A, D1118H | Post-vaccine infection | SARS-COV-2 (COVID-19) - PFIZER | ** |
| Patient58 | M | Not Hispanic or Latino | B.1.1.7 | 20I | N501Y, A570D, D614G, P681H, T716I, S982A, D1118H, H69-V70 deletion, Y144 deletion | Post-vaccine infection | SARS-COV-2 (COVID-19) - PFIZER | ** |
| Patient59 | F | Not Hispanic or Latino | B.1.1.7 | 20I | N501Y, A570D, D614G, P681H, T716I, H69-V70 deletion, Y144 deletion | Post-vaccine infection | SARS-COV-2 (COVID-19) - JANSSEN (J&J) | ** |
| Patient60 | NA | NA | B.1.1.7 | 20I | N501Y, A570D, D614G, P681H, T716I, I870V, S982A, D1118H, H69-V70 deletion, Y144 deletion | Hospitalized for COVID-19 | NA | NA |
| Patient61 | F | Hispanic or Latino | B.1.1.7 | 20I | N501Y, A570D, D614G, P681H, T716I, H69-V70 deletion, Y144 deletion | Post-vaccine infection | SARS-COV-2 (COVID-19) - PFIZER | ** |
| Patient62 | F | Not Hispanic or Latino | B.1.1.7 | 20I | N501Y, A570D, D614G, P681H, T716I, H69-V70 deletion, Y144 deletion, S704L, S982A, D1118H | Hospitalized for COVID-19 | NA | None |
| Patient63 | F | Not Hispanic or Latino | B.1.1.7 | 20I | N501Y, A570D, D614G, P681H, T716I, S982A, D1118H, H69-V70 deletion, Y144 deletion | Hospitalized for COVID-19 | NA | None |
| Patient64 | M | Not Hispanic or Latino | B.1.1.7 | 20I | F4X, N501Y, A570D, D614G, P681H, T716I, S982A, D1118H, H69-V70 deletion, Y144 deletion | Hospitalized for COVID-19 | SARS-COV-2 (COVID-19) - MODERNA | * |
| Patient65 | F | Not Hispanic or Latino | B.1.1.7 | 20I | N501Y, A570D, D614G, P681H, T716I, H69-V70 deletion, Y144 deletion, F86X, S982A, D1118H | Hospitalized for COVID-19 | NA | None |
| Patient66 | M | Hispanic or Latino | B.1.1.519 | 20B | D614G, P681H, T478K, T732A | Hospitalized for COVID-19 | NA | None |
| Patient67 | F | Not Hispanic or Latino | B.1.1.7 | 20I | N501Y, A570D, D614G, P681H, T716I, H69-V70 deletion, Y144 deletion, S982A, D1118H, K1191N | Post-vaccine infection | SARS-COV-2 (COVID-19) - PFIZER | ** |
| Patient68 | M | Not Hispanic or Latino | B.1.1.7 | 20I | N501Y, A570D, D614G, P681H, T716I, H69-V70 deletion, Y144 deletion | Post-vaccine infection | SARS-COV-2 (COVID-19) - MODERNA | ** |
| Patient69 | F | Not Hispanic or Latino | B.1.1.7 | 20I | N501Y, A570D, D614G, P681H, T716I, H69-V70 deletion, Y144 deletion | Hospitalized for COVID-19 | NA | None |
| Patient70 | M | Not Hispanic or Latino | B.1.1.7 | 20I | S494P, N501Y, A570D, D614G, P681H, T716I, H69-V70 deletion, Y144 deletion | Hospitalized for COVID-19 | NA | None |
| Patient71 | M | Not Hispanic or Latino | B.1.1.7 | 20I | N501Y, A570D, D614G, P681H, T716I, H69-V70 deletion, Y144 deletion, F86X, N87X, S982A, D1118H | Hospitalized for COVID-19 | NA | None |
| Patient72 | NA | NA | B.1.1.7 | 20I | N501Y, A570D, D614G, P681H, T716I, H69-V70 deletion, Y144 deletion | Hospitalized for COVID-19 | NA | NA |
| Patient73 | F | Not Hispanic or Latino | P.1 | 20J | K417T, E484K, N501Y, D614G | Post-vaccine infection | SARS-COV-2 (COVID-19) - JANSSEN (J&J) | ** |
| Patient74 | NA | NA | B.1.429 | 20C | L452R, D614G, S13I, W152C | Post-vaccine infection | NA | NA |
| Patient75 | F | Not Hispanic or Latino | B.1.1.7 | 20I | N501Y, A570D, D614G, P681H, T716I, H69-V70 deletion, Y144 deletion | Post-vaccine infection | SARS-COV-2 (COVID-19) - PFIZER | ** |
| Patient76 | M | Not Hispanic or Latino | B.1.1.7 | 20I | F4X, N501Y, A570D, D614G, P681H, T716I, S982A, D1118H, K1191N, H69-V70 deletion, Y144 deletion | Hospitalized for COVID-19 | NA | None |
| Patient77 | F | Not Hispanic or Latino | B.1.351 | 20H | K417N, E484K, N501Y, D614G, A701V, L18F, D80A, F86X, D215G, L242H, A243-, L244-, H245- | Hospitalized for COVID-19 | NA | None |
| Patient78 | M | Not Hispanic or Latino | B.1.1.7 | 20I | N501Y, A570D, D614G, P681H, T716I, H69-V70 deletion, Y144 deletion, S982A, D1118H, P85-, F86-, N87-, D88-, G89-, V90- | Hospitalized for COVID-19 | NA | None |
| Patient79 | F | Not Hispanic or Latino | B.1.1.7 | 20I | N501Y, A570D, D614G, P681H, T716I, H69-V70 deletion, Y144 deletion, L5X, S982A, D1118H | Post-vaccine infection | SARS-COV-2 (COVID-19) - PFIZER | ** |
| Patient80 | F | Not Hispanic or Latino | B.1.1.7 | 20I | N501Y, A570D, D614G, P681H, T716I, H69-V70 deletion, Y144 deletion, S94F, S982A, D1118H | Hospitalized for COVID-19 | NA | None |
| Patient81 | M | Not Hispanic or Latino | B.1.1.7 | 20I | N501Y, A570D, D614G, P681H, T716I, S982A, D1118H, H69-V70 deletion, Y144 deletion | Post-vaccine infection | SARS-COV-2 (COVID-19) - PFIZER | ** |
| Patient82 | F | Not Hispanic or Latino | B.1.1.7 | 20I | N501Y, A570D, D614G, P681H, T716I, S982A, D1118H, H69-V70 deletion, Y144 deletion | Hospitalized for COVID-19 | NA | None |
| Patient83 | M | Not Hispanic or Latino | B.1.1.7 | 20I | N501Y, A570D, D614G, P681H, T716I, H69-V70 deletion, Y144 deletion | Hospitalized for COVID-19 | NA | None |
| Patient84 | NA | NA | B.1.1.7 | 20I | N501Y, A570D, D614G, P681H, T716I, H69-V70 deletion, Y144 deletion | Hospitalized for COVID-19 | NA | NA |
| Patient85 | M | Not Hispanic or Latino | B.1.1.7 | 20I | N501Y, A570D, D614G, P681H, T716I, H69-V70 deletion, Y144 deletion | Hospitalized for COVID-19 | NA | None |
| Patient86 | NA | NA | B.1.1.7 | 20I | N501Y, A570D, D614G, P681H, T716I, H69-V70 deletion, Y144 deletion | Post-vaccine infection | NA | NA |
| Patient87 | NA | NA | B.1.1.7 | 20I | N501Y, A570D, D614G, P681H, T716I, S982A, D1118H, H69-V70 deletion, Y144 deletion | Hospitalized for COVID-19 | NA | NA |
| Patient88 | M | Not Hispanic or Latino | B.1.1.7 | 20I | N501Y, A570D, D614G, P681H, T716I, H69-V70 deletion, Y144 deletion | Post-vaccine infection | SARS-COV-2 (COVID-19) - PFIZER | * |
| Patient89 | F | Not Hispanic or Latino | B.1.1.7 | 20I | N501Y, A570D, D614G, P681H, T716I, S982A, D1118H, H69-V70 deletion, Y144 deletion | Post-vaccine infection | SARS-COV-2 (COVID-19) - JANSSEN (J&J) | ** |
| Patient90 | M | Not Hispanic or Latino | B.1.1.7 | 20I | F86X, N501Y, A570D, D614G, P681H, T716I, S982A, D1118H, H69-V70 deletion, Y144 deletion | Hospitalized for COVID-19 | SARS-COV-2 (COVID-19) - MODERNA | * |
| Patient91 | NA | NA | B.1.351.3 | 20H | K417N, E484K, N501Y, D614G, A701V | Hospitalized for COVID-19 | NA | NA |
| Patient92 | NA | NA | B.1.351 | 20H | K417N, E484K, N501Y, D614G, A701V, L18F, D80A, D215G, L242H, P384L, A243-, L244-, H245- | Post-vaccine infection | SARS-COV-2 (COVID-19) - PFIZER | ** |
| Patient93 | F | Not Hispanic or Latino | P.1 | 20J | K417T, E484K, N501Y, D614G, L18F, T20N, P26S, D138Y, R190S, H655Y, T1027I, V1176F | Hospitalized for COVID-19 | NA | None |
| Patient94 | NA | NA | B.1.1.7 | 20I | N501Y, A570D, D614G, P681H, T716I, H69-V70 deletion, Y144 deletion, S982A, D1118H | Post-vaccine infection | SARS-COV-2 (COVID-19) - PFIZER | ** |
| Patient95 | NA | NA | B.1.526.1 | 20C | L452R, D614G, Y144 deletion | Post-vaccine infection | SARS-COV-2 (COVID-19) - PFIZER | * |
| Patient96 | NA | NA | B.1.1.7 | 20I | F86X, N501Y, A570D, D614G, P681H, T716I, S982A, D1118H, H69-V70 deletion, Y144 deletion | Hospitalized for COVID-19 | NA | NA |
| Patient97 | NA | NA | B.1.565 | 20A | D614G, Q677H, None | Post-vaccine infection | NA | NA |
| Patient98 | NA | NA | B.1.1.7 | 20I | N501Y, A570D, D614G, P681H, T716I, H69-V70 deletion, Y144 deletion, S982A, D1118H | Hospitalized for COVID-19 | NA | NA |
| Patient99 | F | Not Hispanic or Latino | B.1.1.7 | 20I | N501Y, A570D, D614G, P681H, T716I, H69-V70 deletion, Y144 deletion | Hospitalized for COVID-19 | NA | None |
| Patient100 | M | Not Hispanic or Latino | B.1.1.7 | 20I | V90X, N501Y, A570D, D614G, P681H, T716I, S982A, D1118H, H69-V70 deletion, N87-, D88-, G89-, Y144 deletion | Hospitalized for COVID-19 | NA | None |
| Patient101 | F | Unknown | B.1.1.7 | 20I | N501Y, A570D, D614G, P681H, T716I, H69-V70 deletion, Y144 deletion, S982A, D1118H | Hospitalized for COVID-19 | NA | None |
| Patient102 | NA | NA | B.1.617.2 | 21A | T19R, F86X, G142D, E156G, L452R, T478K, D614G, P681R, D950N, F157-, R158- | Post-vaccine infection | NA | NA |

**Table S4:** Clinical characteristics of patients with SARS-CoV-2 genomes sequenced in the Mayo Clinic database which have EHR data available. Comorbidities for each patient were determined from neural network models applied to the clinical notes over the past 5 years leading up to the first sample collection date. Similarly, complications for each patient were determined from neural network models applied to the clinical notes +/- 30 days relative to the first sample collection date. For continuous covariates such as age, mean and standard deviation are shown instead of patient counts and percentages. Clinical characteristics are shown for the following cohorts: (1) All sequenced patients, (2) Sequenced patients with SARS-CoV-2 infection and at least 1 amino acid deletion in position 85 - 90, and (3) Sequenced patients with SARS-CoV-2 infection and without an amino acid deletion in position 85 - 90.

| **Clinical characteristic** | **All patients with deletion mutations** | **Patients with deletions in position 85-90** | **Patients without deletions in position 85-90** |
| --- | --- | --- | --- |
| Total patient count | 71 | 5 | 66 |
| Age at time of sample collection (in years)   - Mean - Standard deviation | 54.1  16.7 | 62.2  3.6 | 53.5  17.2 |
| Sex   - Female - Male | 46 (64.8%)  25 (35.2%) | 2 (40.0%)  3 (60.0%) | 44 (66.7%)  22 (33.3%) |
| Race   - Black / African American - Native American - White / Caucasian - Other - Unknown | 3 (4.2%)  1 (1.4%)  63 (88.7%)  3 (4.2%)  1 (1.4%) | 0 (0%)  0 (0.0%)  5 (100.0%)  0 (0.0%)  0 (0.0%) | 3 (4.5%)  1 (1.5%)  58 (87.9%)  3 (4.5%)  1 (1.5%) |
| Ethnicity   - Hispanic or Latino - Not Hispanic or Latino - Unknown | 1 (1.4%)  69 (97.2%)  1 (1.4%) | 0 (0%)  5 (100.0%)  0 (0.0%) | 1 (1.5%)  64 (97.0%)  1 (1.5%) |
| Comorbidities (within 5 years before sample collection date) |  |  |  |
| - Anemia | 21 (29.6%) | 1 (20.0%) | 20 (30.3%) |
| - Asthma | 23 (32.4%) | 1 (20.0%) | 22 (33.3%) |
| - Cancer | 27 (38.0%) | 1 (20.0%) | 26 (39.4%) |
| - Cardiomyopathy | 7 (9.9%) | 0 (0.0%) | 7 (10.6%) |
| - Chronic kidney disease | 15 (21.1%) | 0 (0.0%) | 15 (22.7%) |
| - Chronic obstructive pulmonary disease | 11 (15.5%) | 1 (20.0%) | 10 (15.2%) |
| - Coronary Artery Disease | 9 (12.7%) | 0 (0.0%) | 9 (13.6%) |
| - HIV | 0 (0.0%) | 0 (0.0%) | 0 (0.0%) |
| - Heart failure | 10 (14.1%) | 0 (0.0%) | 10 (15.2%) |
| - Hyperglycemia | 14 (19.7%) | 1 (20.0%) | 13 (19.7%) |
| - Hypertension | 18 (25.4%) | 1 (20.0%) | 17 (25.8%) |
| - Liver Disease | 9 (12.7%) | 1 (20.0%) | 8 (12.1%) |
| - Neurologic Conditions | 2 (2.8%) | 0 (0.0%) | 2 (3.0%) |
| - Obstructive sleep apnea | 15 (21.1%) | 1 (20.0%) | 14 (21.2%) |
| - Pediatrics | 0 (0.0%) | 0 (0.0%) | 0 (0.0%) |
| - Type 1 diabetes mellitus | 8 (11.3%) | 0 (0.0%) | 8 (12.1%) |
| - Type 2 diabetes mellitus | 16 (22.5%) | 0 (0.0%) | 16 (24.2%) |
| Complications (+/- 30 days relative to sample collection date) |  |  |  |
| - ARD ALI | 13 (18.3%) | 2 (40.0%) | 11 (16.7%) |
| - Acute kidney injury | 12 (16.9%) | 1 (20.0%) | 11 (16.7%) |
| - Anemia | 20 (28.2%) | 1 (20.0%) | 19 (28.8%) |
| - Cardiac arrest | 1 (1.4%) | 0 (0.0%) | 1 (1.5%) |
| - Cardiac arrhythmias | 29 (40.8%) | 2 (40.0%) | 27 (40.9%) |
| - Chronic fatigue syndrome | 1 (1.4%) | 0 (0.0%) | 1 (1.5%) |
| - Disseminated intravascular coagulation | 1 (1.4%) | 0 (0.0%) | 1 (1.5%) |
| - Encephalopathy Delirium | 7 (9.9%) | 0 (0.0%) | 7 (10.6%) |
| - Heart failure | 8 (11.3%) | 0 (0.0%) | 8 (12.1%) |
| - Hyperglycemia | 15 (21.1%) | 1 (20.0%) | 14 (21.2%) |
| - Hypertension | 13 (18.3%) | 1 (20.0%) | 12 (18.2%) |
| - Myocardial infarction | 3 (4.2%) | 0 (0.0%) | 3 (4.5%) |
| - Numbness | 7 (9.9%) | 1 (20.0%) | 6 (9.1%) |
| - Pleural effusion | 7 (9.9%) | 1 (20.0%) | 6 (9.1%) |
| - Pulmonary embolism | 8 (11.3%) | 1 (20.0%) | 7 (10.6%) |
| - Respiratory failure | 12 (16.9%) | 2 (40.0%) | 10 (15.2%) |
| - Sepsis | 7 (9.9%) | 1 (20.0%) | 6 (9.1%) |
| - Septic shock | 3 (4.2%) | 0 (0.0%) | 3 (4.5%) |
| - Stroke | 2 (2.8%) | 0 (0.0%) | 2 (3.0%) |
| - Venous thromboembolism | 10 (14.1%) | 2 (40.0%) | 8 (12.1%) |

**Table S5:** List of GISAID accession IDs with one or more deletions observed in the 85-90 region of the Spike protein.

| **Accession ID** | **Collection date** | **Location** |
| --- | --- | --- |
| EPI_ISL_454256 | 4/7/20 | Europe / Portugal |
| EPI_ISL_1034182 | 12/26/20 | Asia / India / Goa |
| EPI_ISL_1236478 | 2/26/21 | North America / USA / Texas / Houston |
| EPI_ISL_1286100 | 3/6/21 | Europe / Germany / Hamburg |
| EPI_ISL_1309764 | 2/15/21 | Europe / United Kingdom / England |
| EPI_ISL_1493068 | 1/25/21 | North America / USA / Oregon / Washington County |
| EPI_ISL_1725555 | 3/10/21 | Europe / Germany / Berlin |
| EPI_ISL_1820843 | 4/19/21 | Europe / Netherlands / Noord-Brabant |
| EPI_ISL_1841248 | 4/18/21 | Asia / India / Gujarat / Ahmedabad |
| EPI_ISL_1840693 | 4/17/21 | North America / USA / Texas |
| EPI_ISL_1844630 | 4/20/21 | Europe / Germany / Bavaria |
| EPI_ISL_2023473 | 5/8/21 | North America / USA / Oregon |
| EPI_ISL_2039575 | 4/16/21 | North America / USA / New Jersey |
| EPI_ISL_2106199 | 1/21/21 | Europe / Austria / East Tyrol |
| EPI_ISL_2254264 | 4/18/21 | North America / USA / Michigan |
| EPI_ISL_2264501 | 5/8/21 | Europe / Germany / Saarland |
| EPI_ISL_2377899 | 4/29/21 | North America / USA / Ohio |
| EPI_ISL_2388039 | 5/21/21 | Europe / Germany / Baden-Wurttemberg |
| EPI_ISL_2391848 | 5/21/21 | Europe / Spain / Catalunya |
| EPI_ISL_2438547 | 5/26/21 | Asia / Pakistan / Azad Kashmir |
| EPI_ISL_2510028 | 3/19/21 | Europe / France / Auvergne-Rhone-Alpes / Grenoble |
| EPI_ISL_2550761 | 5/18/21 | North America / USA / Maryland / MDH |
| EPI_ISL_2557914 | 5/26/21 | North America / USA / Oregon / Jackson County |
| EPI_ISL_2589764 | 12/27/20 | Europe / Finland |
| EPI_ISL_2650478 | 12/9/20 | Europe / Poland / Zachodniopomorskie |
| EPI_ISL_2652049 | 5/20/21 | Europe / Spain / Catalunya |
| EPI_ISL_2652052 | 5/20/21 | Europe / Spain / Catalunya |
| EPI_ISL_2652055 | 5/20/21 | Europe / Spain / Catalunya |
| EPI_ISL_2657362 | 6/23/21 | Europe / Spain / Catalunya |
| EPI_ISL_2681437 | 4/12/21 | Europe / North Macedonia / Gevgelija |
| EPI_ISL_2689086 | 6/11/21 | North America / USA / Maryland |
| EPI_ISL_2765557 | 6/24/21 | Europe / Italy / Sicilia |
| EPI_ISL_2761960 | 6/18/21 | Europe / Germany / Hesse |

**Table S6: Summary of literature on experimental data supporting observations.** Experimental findings have corroborated the effect of three out of five (other than the new RDRs proposed by us in this study) RDRs on the increased infectivity and/or reduced neutralization of SARS-CoV-2 Spike protein with respect to the wild type Spike.

| **Key findings** | **Mutations studied** | **Experimental method** | **Reference** |
| --- | --- | --- | --- |
| Impact of Lambda variant (previously designated as “Chile variant”) on immune escape and infectivity. | Mutations in Lambda variant (specific effects of 246-252 deletion were not studied) | Neutralization assay: Geometric mean titre of ID_50_ (50% inhibitory dilution) titres decreased by a factor of 3.05, 2.33, 2.03 with respect to that of the wild type Spike for the Lambda, Gamma and Alpha Spikes, respectively. | Acevedo et al., 2021[^20^](https://paperpile.com/c/9ggqQZ/Bgvd) |
| Δ85-90 is part of a linear epitope | Residues 79-93 in Spike | High-density microarray: p-value from unpaired t-test to discriminate fluorescence intensity of IgG immune-reactivity of serum from COVID-19-positive and healthy patients. | Musicò et al., 2021[^26^](https://paperpile.com/c/9ggqQZ/iP5b) |
| Reduced neutralization of Kappa and Delta variants | Mutations in the Kappa and Delta variants | Live-virus focus reduction neutralization assay (FRNT): Kappa and Delta variants are 6.8-fold and 2.9-fold less susceptible to neutralization by convalescent and vaccinated sera, respectively, as compared to the wild type. | Edara et al., 2021[^22^](https://paperpile.com/c/9ggqQZ/OMPr) |
| Reduced neutralization of Delta variant due to reduced / loss of binding to antibodies (monoclonal antibodies, Bamlanivimab, Etesevimab, Casirivimab, Imdevimab, anti-RBD, anti-NTD) | Mutations in Delta variant (specific effects of ΔF157/ΔR158 were not studied) | S-Fuse assay: IC_50_ values for Delta with respect to the D614G Spike were examined for all antibodies. | Planas et al., 2021[^21^](https://paperpile.com/c/9ggqQZ/2mCD) |
| ΔH69/ΔV70 increases Spike incorporation, infectivity and syncytium formation | ΔH69, ΔV70 | Infectivity assay: Two-fold increase in viral infectivity of ΔH69/ΔV70 mutant compared with the wild type in stable ACE2-expressing HeLa cervical epithelial cells. | Meng et al., 2021[^35^](https://paperpile.com/c/9ggqQZ/WVie) |
| ΔH69/ΔV70 increases infectivity of the Spike protein | ΔH69, ΔV70 | Pseudotyping assay: Two-fold higher infectivity of theΔH69/ΔV70 mutant compared to that of the wild type. | Kemp et al., 2021[^36^](https://paperpile.com/c/9ggqQZ/0TaW) |
| Reduced neutralization of the Alpha variant to monoclonal antibodies | ΔH69, ΔV70, ΔY144 | Focus reduction neutralization assay (FRNT): 5.7-fold reduction in FRNT_50_ value for NTD-binding monoclonal antibody for Alpha Spike with respect to wild type Spike. | Supasa et al., 2021[^23^](https://paperpile.com/c/9ggqQZ/NM6S) |
| Immune escape in Beta variant (lack of neutralization by human monoclonal antibodies due to NTD deletion) | 242-244 deletion | Focus reduction neutralization assay (FRNT): 13.3-fold reduction in neutralization titres for Beta variant with respect to the wild type. | Zhou et al., 2021[^24^](https://paperpile.com/c/9ggqQZ/2tNK) |
| Increased infectivity in Spike protein with deletion in cytoplasmic tail | SΔCT13 (13 amino acid deletion in the cytoplasmic tail) | Pseudovirus neutralization assay: Significantly increased viral entry in HEK293T-human ACE2 cell for the cytoplasmic tail deletion mutant with respect to the wild type Spike. | Yu et al., 2021[^37^](https://paperpile.com/c/9ggqQZ/nBtO) |
